# Carbohydrate-active enzymes in *Trichoderma harzianum*: a bioinformatic analysis bioprospecting for key enzymes for the biofuels industry

**DOI:** 10.1186/s12864-017-4181-9

**Published:** 2017-10-12

**Authors:** Jaire Alves Ferreira Filho, Maria Augusta Crivelente Horta, Lilian Luzia Beloti, Clelton Aparecido dos Santos, Anete Pereira de Souza

**Affiliations:** 1 Center for Molecular Biology and Genetic Engineering (CBMEG), University of Campinas (UNICAMP), Campinas, SP Brazil; 2 Graduate Program in Genetics and Molecular Biology, Institute of Biology, UNICAMP, Campinas, SP Brazil; 3 Department of Plant Biology, Institute of Biology, UNICAMP, Campinas, SP Brazil

**Keywords:** *Trichoderma harzianum*, CAZymes, Glycoside hydrolases, Phylogeny, RNA-Seq, Cellulases

## Abstract

**Background:**

*Trichoderma harzianum* is used in biotechnology applications due to its ability to produce powerful enzymes for the conversion of lignocellulosic substrates into soluble sugars. Active enzymes involved in carbohydrate metabolism are defined as carbohydrate-active enzymes (CAZymes), and the most abundant family in the CAZy database is the glycoside hydrolases. The enzymes of this family play a fundamental role in the decomposition of plant biomass.

**Results:**

In this study, the CAZymes of *T. harzianum* were identified and classified using bioinformatic approaches after which the expression profiles of all annotated CAZymes were assessed via RNA-Seq, and a phylogenetic analysis was performed. A total of 430 CAZymes (3.7% of the total proteins for this organism) were annotated in *T. harzianum*, including 259 glycoside hydrolases (GHs), 101 glycosyl transferases (GTs), 6 polysaccharide lyases (PLs), 22 carbohydrate esterases (CEs), 42 auxiliary activities (AAs) and 46 carbohydrate-binding modules (CBMs). Among the identified *T. harzianum* CAZymes, 47% were predicted to harbor a signal peptide sequence and were therefore classified as secreted proteins. The GH families were the CAZyme class with the greatest number of expressed genes, including GH18 (23 genes), GH3 (17 genes), GH16 (16 genes), GH2 (13 genes) and GH5 (12 genes). A phylogenetic analysis of the proteins in the AA9/GH61, CE5 and GH55 families showed high functional variation among the proteins.

**Conclusions:**

Identifying the main proteins used by *T. harzianum* for biomass degradation can ensure new advances in the biofuel production field. Herein, we annotated and characterized the expression levels of all of the CAZymes from *T. harzianum,* which may contribute to future studies focusing on the functional and structural characterization of the identified proteins.

**Electronic supplementary material:**

The online version of this article (10.1186/s12864-017-4181-9) contains supplementary material, which is available to authorized users.

## Background

Brazil harbors a highly diverse fungal community and many of these species are of great biotechnological relevance [1]. Fungal species produce important enzyme classes that play a key role in the decomposition of organic materials, particularly those derived from plants. These enzymes can be applied in various industrial areas [2], such as the production of biofuels from vegetal biomass. Cellulose is a major component of the cell wall of plants. This structural polysaccharide is a complex polymer, and only a specific set of enzymes can breakdown cellulose [3]. Many of these enzymes are produced by fungi, such as those of the genera *Aspergillus*, *Neurospora* and *Trichoderma*.

*Trichoderma harzianum* is a filamentous fungus [4] and a recognized biocontrol agent that is effective against phytopathogenic fungi [5]. *T. reesei* is the best-studied cellulolytic fungus for which there are available resolved crystallographic structures among enzymes of the cellulolytic complex [6, 7]. However, *T. harzianum* also produces enzymes capable of hydrolyzing and metabolizing the cellulose present in plant biomass [8, 9].

Studies on *T. harzianum* strains show that they are able to produce a cellulolytic complex with higher beta-glucosidase activity than that shown by *T. reesei* [8, 10]. Furthermore, significant endoglucanase, xylanase and cellobiohydrolase activities have also been detected [11]. Although the enzymes produced by *T. harzianum* harbor great biotechnological potential, most studies in this species have been directed toward the area of biological control [12, 13]. Thus, there is a shortage of studies exploring genomic data related to its capacity to degrade biomass and the regulation and expression of the genes involved in biodegradation.

Enzymes active in carbohydrate metabolism are defined in the international literature as carbohydrate-active enzymes (CAZymes) [14]. The families of enzymes that compose this group can be found in the CAZy database (www.cazy.org). The protein families in the CAZy database are grouped into five different classes, as follows: glycoside hydrolases (GHs); glycosyl transferases (GTs); polysaccharide lyases (LPs); carbohydrate esterases (CEs); and auxiliary activities (AAs). The most abundant family in the CAZy database is the GHs; the enzymes in this family play a fundamental role in the decomposition of plant biomass and have therefore been the target of several studies on enzymatic hydrolysis [15, 16]. Recent studies have revealed the importance of the AA family as aids in the process of cellulose degradation [17].

In this work, we used the available structural genomic data from *T. harzianum* T6776 [18] to perform a complete functional annotation of the CAZyme content. In addition, we employed the data generated from a previous RNA-Seq study [19] to analyze the expression of this set of genes. To investigate the functional diversity of these proteins, phylogenetic analyses of the AA9/GH61, CE5 and GH55 families were performed. Based on our results, we delineated specific CAZyme clusters that act on biomass substrates for depolymerization. These data should contribute to the search for more efficient enzymatic systems for the biomass degradation process and for the functional and heterologous expression of important proteins with hydrolytic activity.

## Methods

### Data sources

The nucleotide and protein sequences of *T. harzianum* T6776 - Th (PRJNA252551), *T. reesei* RUT C-30 - Tr (PRJNA207855), *T. atroviride* IMI 206040 - Ta (PRJNA19867) and *T. virens* Gv29–8 - Tv (PRJNA19983) were downloaded from the NCBI database (www.ncbi.nlm.nih.gov). The *T. harzianum* IOC-3844 RNA-Seq reads are available in the NCBI Sequence Read Archive (SRA) under accession number PRJNA175485.

### Functional annotation of CAZymes

Information derived from the CAZy database [14] was downloaded for each CAZyme family (www.cazy.org). The protein sequences of *T. harzianum*, *T. reesei*, *T. atroviride* and *T. virens* were used as queries in BLASTp (Basic local alignment search tool) searches against the locally built CAZy BLAST database. Only blast matches showing an e-value less than 10^−11^, identity greater than 30% and queries covering greater than 70% of the sequence length were retained and classified according CAZyme catalytic group as GHs, GTs, PLs, CEs, CBMs or AAs (Additional files 1, 2, 3 and 4).

Annotations were performed with Blast2Go [20] using BLASTx. All of the protein sequences of the *T. harzianum* CAZymes were functionally annotated based on homology. The *T. harzianum* CAZymes were further aligned to the PFAM profiles [21] of the families through a search of the Conserved Domain Database (CDD) of NCBI [22] and a search against the PFAM v28.0 database. InterPro protein domains were predicted using InterProScan (http://www.ebi.ac.uk/interpro/) [23].

Signal peptides of the *T. harzianum* CAZymes were predicted using SignalP v.4.1 (http://www.cbs.dtu.dk/services/SignalP/) [24] with default parameters, and all of the proteins with signal peptides were analyzed for the presence of transmembrane domains using the web server TMHMM v.2.0 (http://www.cbs.dtu.dk/services/TMHMM/) [25] with default parameters. TargetP v.1.1 (http://www.cbs.dtu.dk/services/TargetP/) [26] with the following parameters: organism group – “Non-plant” and cutoffs – default, and Cello v.2.5 (http://cello.life.nctu.edu.tw/) [27] with default parameters, were employed for the prediction of subcellular localization.

### Transcriptional analysis of *T. harzianum* CAZymes

The expression levels of the *T. harzianum* CAZymes were analyzed using RNA-Seq data obtained from a previous study [19] in which the transcripts were obtained following growth of the fungus on three different carbon sources, lactose (LAC), cellulose (CEL), and delignified sugarcane bagasse (DSB), to induce mycelial growth. The quality control of the reads were as follows: quality limit - 0.03; ambiguous limit - 2; minimum final number of nucleotides in read - 65; and phred scale - 15 [19].

The reads from the RNA-Seq library were mapped against the *T. harzianum* CAZymes using the CLC Genomics Workbench (CLC bio – v9.0; Finlandsgade, Dk) [28] with the following parameters: mapping settings (minimum length fraction = 0.9, minimum similarity fraction = 0.8, and maximum number of hits for a read = 15) and paired settings (minimum distance = 180 and maximum distance = 250, including the broken pairs counting scheme). The expression values were expressed in reads per kilobase of exon model per million mapped reads (RPKM) and the normalized value for each sample was calculated in transcripts per million (TPM) [29] according to the following formula:

$$ {\boldsymbol{TPM}}_{\boldsymbol{i}}=\left[\frac{{\boldsymbol{RPKM}}_{\boldsymbol{i}}}{\sum_{\boldsymbol{j}}{\boldsymbol{RPKM}}_{\boldsymbol{j}}}\right]\times {10}^6 $$

where *RPKM*_*i*_ is the expression value for each gene in a sample and *∑*_*j*_ *RPKM*_*j*_ is the sum of the RPKM values of all genes in a sample. A total of the TPM expression values was calculated for each GH family according to the summing the expression level of all the genes that were identified for that particular family. A hierarchical clustering analysis was conducted with CLC bio using the single linkage method and Euclidian distance according to the cluster features of the log2-transformed expression values.

### Phylogenetic analysis of CAZymes

The CAZyme sequences from the AA9/GH61, CE5 and GH55 families from *T. harzianum*, *T. reesei*, *T. atroviride*, *T. virens* and ten other species of fungi (Additional file 5) were used as the basis for constructing the phylogenetic trees. The sequences were aligned using ClustalW [30], implemented in the Molecular Evolutionary Genetics Analysis (MEGA) software version 7.0 [31] with the following parameters: gap opening penalties of 10 and 3 and gap extension penalties of 0.1 and 5 for the pairwise alignment and multiple alignment, respectively. The phylogenetic analyses were performed in MEGA7 using the maximum likelihood (ML) method inference [32] based on the Jones-Taylor-Thornton (JTT) matrix-based model and 1000 bootstrap replicates [33] for each analysis. The initial tree is drawn to scale, obtained automatically by applying the Neighbor-joining and BioNJ algorithms to a matrix of pairwise distances estimated using the JTT model and the topology with superior log likelihood values. Pairwise deletion was employed to address alignment gaps and missing data. The trees were visualized and edited using the Figtree program (http://tree.bio.ed.ac.uk/software/figtree/).

## Results

### Identification of CAZymes in *T. harzianum*

In this study, protein prediction for *T. harzianum* T6776 was used as a tool to annotate and analyze the total CAZyme content of this fungus. The identified proteins were re-annotated based on their functional classification, protein domains and secretion signal prediction. In addition, an expression analysis was performed by mapping the reads obtained from RNA-Seq under three biological conditions, and three CAZyme families were employed for the analysis of phylogenetic diversity. Using genomic, transcriptomic and phylogenetic association data, this study describes and characterizes the total (and more complete) set of CAZymes for *T. harzianum*, which is a species that presents high potential in prospecting for hydrolytic enzymes for the degradation of lignocellulosic biomass.

**Fig. 1:**
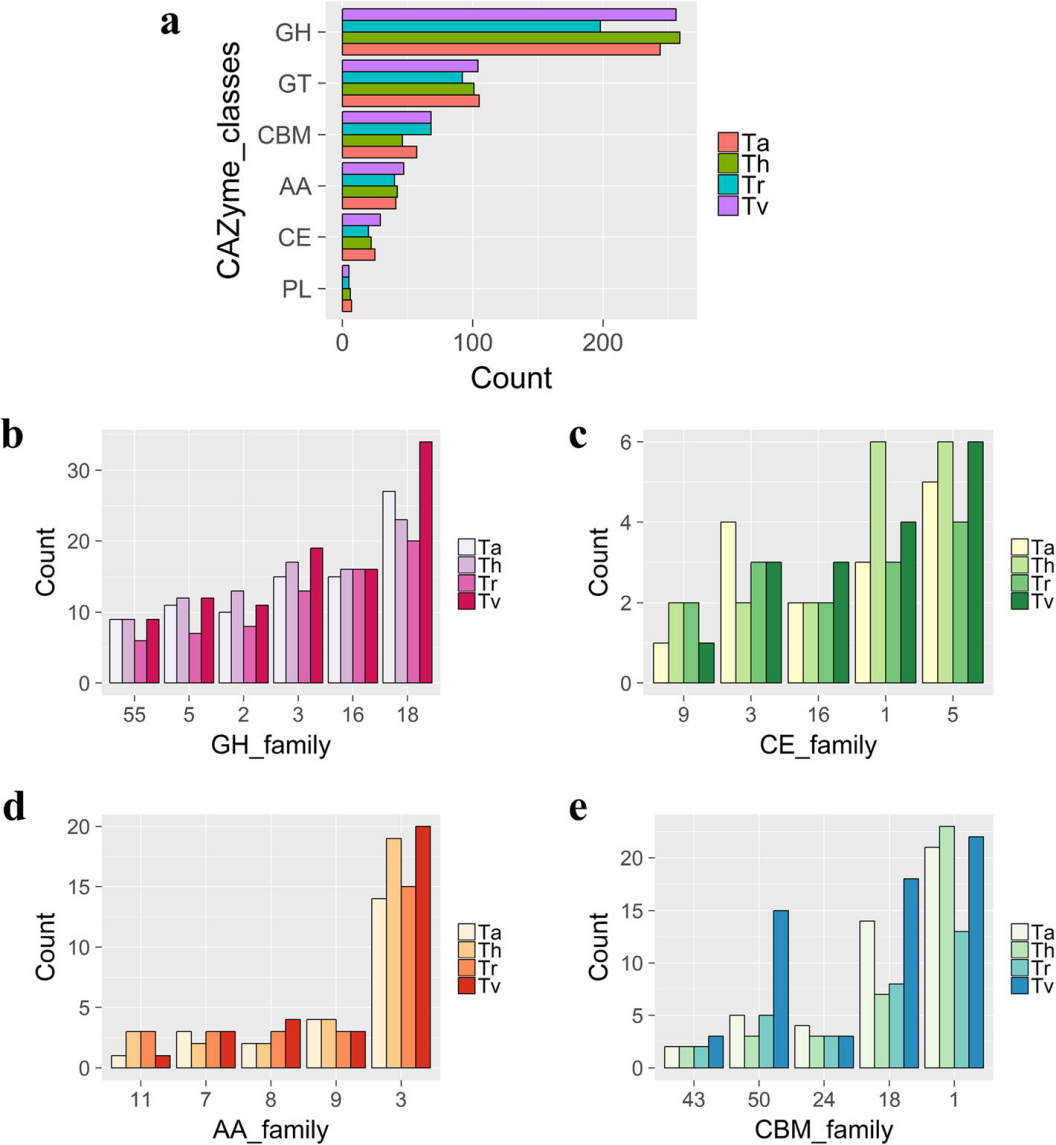
Number of CAZymes in *T. harzianum*, *T. reesei*, *T. virens* and *T. atroviride*. Total of the CAZyme classes for the evaluated species (**a**); number of GH families: GH18, GH16, GH3, GH2, GH5 and GH55 (**b**); number of CE families: CE1, CE5, CE3, CE9 and CE16 (**c**); number of AA families: AA3, AA9, AA11, AA7 and AA8 (**d**); and number of CBM modules: CBM1, CBM18, CBM50, CBM24 and CBM43 (**e**)

The initial set of CAZymes was identified by mapping all of the proteins of *T. harzianum* against the CAZy database using the BLASTp search tool. After removing the proteins that did not meet the filtering criteria, a total of 430 proteins were maintained, which corresponds to 3.7% of the total of 11,498 proteins predicted for this organism [18]. The total protein pool was grouped according to the classification criteria of the CAZy database. Thus, a total of 259 GHs, 101 GTs, 6 PLs, 22 CEs, and 42 AAs as well as an additional 46 proteins that also contained a CBM were identified (Fig. 1 and Tables 1 and Additional file 6: Table S1). A total of 63 GHs families were identified in the *T. harzianum* genome (Table 2).

**Table 1:** Total CAZymes from *T. harzianum* and other fungal species

Species	GH	GT	PL	CE	AA	CBM	Total	Reference
*T. harzianum*	259	101	6	22	42	46	430	This study
*T. reesei* RUT C-30	198	92	5	20	40	35	355	This study
*T. virens*	256	104	5	29	47	68	441	This study
*T. atroviride*	244	105	7	25	41	57	422	This study
*T. reesei* QM6a	201	99	5	22	–	24	327	[39]
*F. graminearum*	253	110	21	45	72	87	501	*
*A. nidulans*	266	91	21	30	33	44	441	*
*N. crassa*	173	78	4	21	35	42	311	*
*S. cerevisiae*	57	73	0	2	5	12	137	*
*A. oryzae*	306	117	23	29	29	37	504	*
*A. niger*	253	121	8	68	68	55	518	*

*CAZy database (www.cazy.org)

**Table 2:** Evaluation of the GH classes in *T. harzianum*

GH family	Number	Principal enzyme activity^a^	EC number^a^	RNA-Seq (TPM)^b^		
				CEL	DSB	LAC
GH1	4	β-glucosidase	3.2.1.21	26,464.6	24,945.6	25,780.2
GH2	13	β-mannosidase	3.2.1.25	10,109.9	10,652.8	10,615.0
GH3	17	β-glucosidase	3.2.1.21	23,132.5	24,232.1	24,991.6
GH4	1	α-glucosidase	3.2.1.22	1405.5	1866.2	1500.3
GH5	12	Endo- β-1,4-glucanase	3.2.1.4	15,970.5	17,669.5	16,084.8
GH6	1	cellobiohydrolase	3.2.1.91	35,003.8	46,112.3	48,148.5
GH7	2	Endo- β-1,4-glucanase	3.2.1.4	56,503.3	62,881.9	73,957.0
GH10	2	Endo-1,4- β-xylanase	3.2.1.8	46,748.0	61,201.9	53,469.7
GH11	4	Endo-1,4- β-xylanase	3.2.1.8	53,279.5	69,046.4	64,929.2
GH12	3	Endo- β-1,4-glucanase	3.2.1.4	10,362.0	12,937.4	10,779.4
GH13	8	α-amylase	3.2.1.1	5028.8	4708.1	4757.5
GH15	2	Glucoamylase	3.2.1.3	3776.3	3476.4	3103.1
GH16	16	Xyloglucanase	3.2.1.151	59,203.1	56,083.1	62,280.8
GH17	4	Endo-1,3- β-glucosidase	3.2.1.39	11,593.9	11,757.2	13,578.5
GH18	23	Chitinase	3.2.1.14	63,673.6	84,188.2	93,866.4
GH20	3	β-hexosaminidase	3.2.1.52	35,521.8	33,517.7	23,889.0
GH23	1	Lysozyme	3.2.1.17	1227.2	849.4	1093.1
GH24	1	Lysozyme	3.2.1.17	2492.6	1450.6	1586.9
GH25	1	Lysozyme	3.2.1.17	1885.9	1657.9	1559.4
GH26	2	β-mannanase	3.2.1.78	314.0	315.2	308.3
GH27	9	α-galactosidase	3.2.1.22	1524.0	1354.3	1373.8
GH28	3	polygalacturonase	3.2.1.15	38,662.7	26,783.2	27,177.1
GH30	6	Endo- β-1,4-xylanase	3.2.1.8	4233.3	3652.1	4332.6
GH31	5	α-glucosidase	3.2.1.20	6161.9	5753.5	7524.8
GH32	2	Invertase	3.2.1.26	0.0	0.0	0.0
GH33	1	neuraminidase	3.2.1.18	0.0	0.0	0.0
GH35	1	β-galactosidase	3.2.1.23	1814.5	1099.7	1961.4
GH36	2	α-galactosidase	3.2.1.22	308.4	255.6	240.4
GH37	2	α-trehalase	3.2.1.28	2379.5	1017.6	1640.6
GH38	1	α-mannosidase	3.2.1.24	964.8	1116.1	960.2
GH39	3	β-xylosidase	3.2.1.37	648.2	444.7	446.0
GH43	5	β-xylosidase	3.2.1.37	3643.7	3004.4	4153.1
GH45	3	Endoglucanase	3.2.1.4	4845.5	4891.1	4353.0
GH47	6	α-mannosidase	3.2.1.113	8278.3	6385.7	7684.9
GH49	1	Dextranase	3.2.1.11	0.0	0.0	0.0
GH54	2	α-L-arabinofuranosidase	3.2.1.55	1253.1	1234.0	2333.9
GH55	9	Endo- β-1,3-glucanase	3.2.1.39	3113.4	2662.3	3444.8
GH62	2	α-L-arabinofuranosidase	3.2.1.55	9919.7	8963.6	14,790.8
GH63	1	α-glucosidase	3.2.1.106	812.5	509.6	521.8
GH64	3	β-1,3-glucanase	3.2.1.39	9176.4	8197.0	8943.6
GH65	1	α-trehalase	3.2.1.28	1585.3	1273.2	1085.3
GH67	2	α-glucuronidase	3.2.1.139	1300.6	732.9	1057.3
GH71	6	α-1,3-glucanase	3.2.1.59	13,633.2	15,112.4	16,804.9
GH72	5	β-1,3-glucanosyltransglycosylase	2.4.1-	26,604.3	25,458.0	26,995.4
GH75	5	Chitosanase	3.2.1.132	3030.0	4812.3	4033.0
GH76	9	α-1,6-mannanase	3.2.1.101	14,462.0	11,978.6	10,448.3
GH78	3	α-L-rhamnosidase	3.2.1.40	111.9	44.6	55.3
GH79	3	β-glucuronidase	3.2.1.31	299.1	232.8	219.4
GH81	2	Endo- β-1,3-glucanase	3.2.1.39	244.0	164.1	137.3
GH88	2	d-4,5-unsaturated β-glucuronyl hydrolase	3.2.1	263.6	100.3	80.0
GH89	2	α-N-acetylglucosaminidase	3.2.1.50	1059.2	760.0	533.5
GH92	8	Mannosyl-oligosaccharide α-1,2-mannosidase	3.2.1.113	7002.9	5376.4	5435.1
GH93	4	Exo- α-L-1,5-arabinanase	3.2.1	7736.1	4164.5	4140.9
GH95	5	α-L-fucosidase	3.2.1.51	680.7	554.0	602.6
GH105	1	Unsaturated rhamnogalacturonyl hydrolase	3.2.1.172	0.0	0.0	0.0
GH114	1	Endo- α-1,4-polygalactosaminidase	3.2.1.109	2290.2	1929.7	1214.4
GH115	1	Xylan α-1,2-glucuronidase	3.2.1.131	3420.7	3848.9	3455.7
GH125	2	Exo- α-1,6-mannosidase	3.2.1	3221.8	2177.3	2401.5
GH127	1	β-L-arabinofuranosidase	3.2.1.185	628.6	399.2	378.0
GH128	4	β-1,3-glucanase	3.2.1.39	5748.1	3170.0	3356.8
GH132	2	Activity on β-1,3-glucan	–	4211.0	5376.7	5835.3
GH133	1	Amylo- α-1,6-glucosidase	3.2.1.33	1480.5	1058.1	1132.8
GH135	2	α-1,4-galactosaminogalactan hydrolase	3.2.1	153.7	134.0	121.9

*TPM,* transcripts per million

^a^CAZy database (www.cazy.org)

^b^The expression values for the different GH families were calculated by summing the level of TPM expression in each gene composing the family

Among the 430 CAZymes identified, 204 (47%) were predicted to harbor a signal peptide and were classified as potential secreted proteins, including 155 GHs, 17 CEs, 8 GTs, 6 PLs and 18 AAs. The proteins that contained a signal peptide were also investigated for the presence of transmembrane domains, resulting in the identification of 19 proteins containing such domains within this group. Searches for mitochondrial proteins were also performed in the CAZymes set, and 37 proteins were identified.

The CAZyme contents of *T. reesei* RUT C-30 were also investigated, and 198 GHs, 92 GTs, 5 PLs, 20 CEs, 40 AAs and 35 CBMs were identified, totaling 355 CAZymes (Table 1 and Additional file 6: Table S1). In *T. virens* and *T. atroviride*, 441 and 422 CAZymes were identified, respectively. The GH18 family, represented mainly by chitinase-like proteins, was the most frequent in the four species, while the GH3 family, which includes enzymes important for the process of cellulose degradation, was the second most frequent in the studied species (Fig. 1).

### Enzymes related to cellulose and hemicellulose degradation in *T. harzianum*

The GHs are the main class of enzymes within the CAZymes responsible for cellulose degradation, and the GH5, GH7, GH12, GH45, GH1, GH3 and GH6 families play particularly important roles (Table 3). Twenty-three CBM1 domains have been identified in *T. harzianum*. Two auxiliary families were identified that are related to the cellulose degradation mechanism in *T. harzianum*: AA8 (2 proteins) and AA9 (4 proteins).

**Table 3:** Cellulolytic enzymes of *Trichoderma* spp

Species	GH5	GH7	GH12	GH45	GH1	GH3	GH6	Total
*T. harzianum*	12	2	3	3	4	17	1	42
*T. reesei*	7	2	2	1	2	13	1	28
*T. atroviride*	11	2	3	1	4	15	1	37
*T. virens*	12	2	4	2	2	19	1	42

The major families involved in the degradation of hemicellulose in *T. harzianum* are GH95, GH67, GH62, GH54, GH43, GH26, GH11, and GH10, and the total number of enzymes, including all families, is 24. The greatest number of enzymes belong to the GH43 and GH95 family (5 proteins) and the fewest to the GH67, GH62, GH54, GH26 and GH10 families (with 2 proteins each) (Table 4).

**Table 4:** Hemicellulose-degrading enzymes of *Trichoderma* spp

Species	GH95	GH67	GH62	GH54	GH43	GH26	GH11	GH10	Total
*T. harzianum*	5	2	2	2	5	2	4	2	24
*T. reesei*	4	1	1	2	2	0	3	1	14
*T. atroviride*	4	2	2	2	6	0	4	1	21
*T. virens*	4	2	3	2	4	2	4	2	23

### Expression analysis using *T. harzianum* RNA-Seq data

**Fig. 2:**
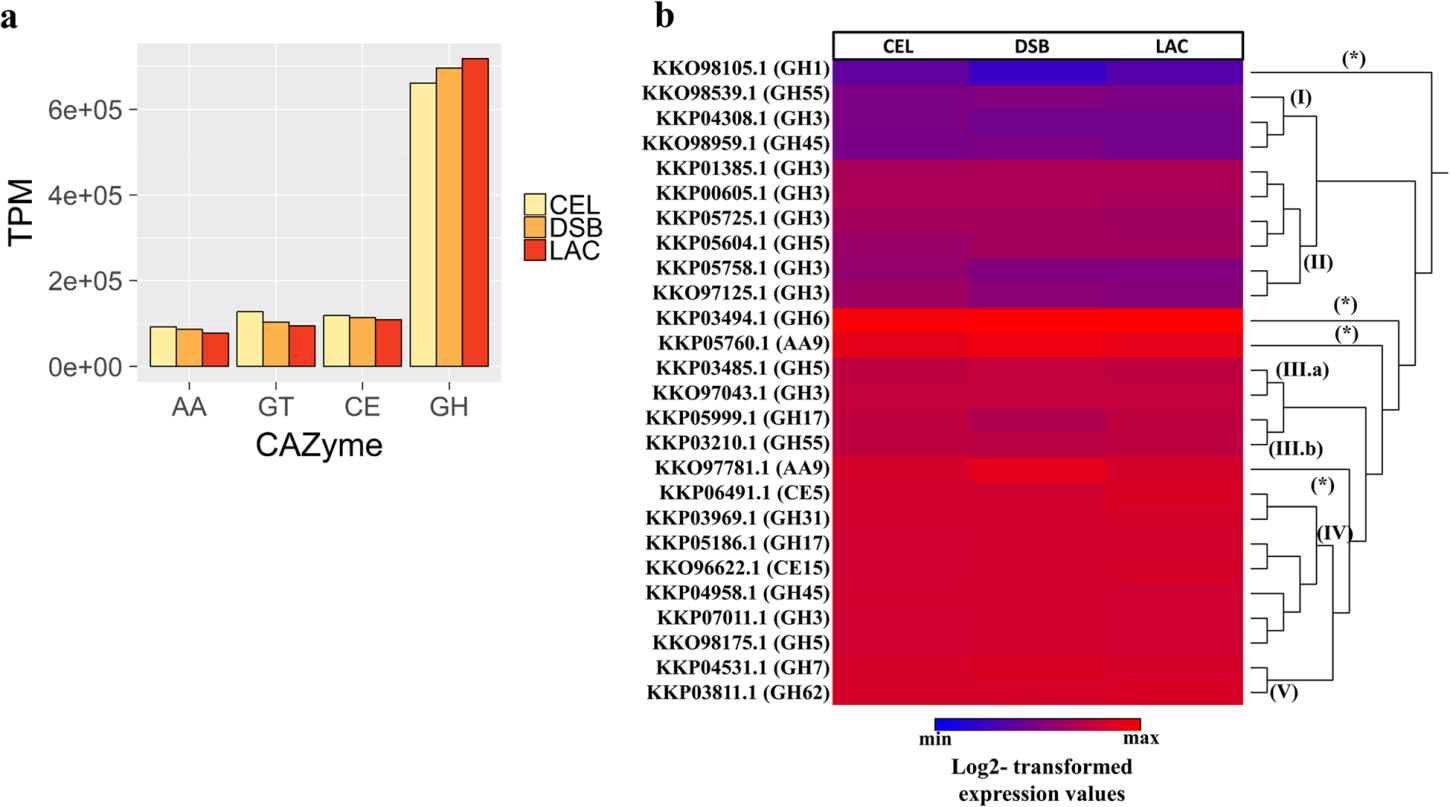
Evaluation of CAZyme expression in *T. harzianum* by means of RNA-Seq. Quantification of the expression of the main CAZyme classes (GH, GT, AA and CE) in TPM (**a**) and hierarchical clustering of the log2-transformed expression values of the 26 CAZymes of *T. harzianum* (**b**). *TPM,* transcripts per million

The relative expression of the 430 CAZymes of *T. harzianum* was studied through computational analysis with the *T. harzianum* IOC3844 RNA-Seq reads (Fig. 2a and Table 2 and Additional file 7: Table S2). The enzymes that exhibited expression greater than zero under the cellulose condition included 400 CAZymes, as follows: 243 GHs, 19 CEs, 4 PLs and 35 AAs. The GH families with the greatest number of expressed genes were GH18 (23 genes – 63,673.6 TPM in CEL), GH3 (17 genes – 11,593.9 TPM in CEL), GH16 (16 genes – 59,203.1 TPM in CEL), GH2 (13 genes – 5028.8 TPM in CEL) and GH5 (12 genes – 15,970.5 TPM in CEL).

The most highly expressed genes were an exoglucanase 1 (CBM1 module) from the GH7 family (KKO99004.1), under conditions of CEL and LAC induction, and an endo-1,4-β-xylanase from the GH10 family (KKP04658.1), under DSB induction. Among the 430 genes, 30 were not expressed in CEL, 17 were not expressed in DSB, and 16 were not expressed in LAC.

The CAZyme genes related to the degradation of cellulose (GH1, GH3, GH5, GH6, GH45 and AA9), hemicellulose (GH31 and GH62), and glucan (GH17 and GH55) as well as esterase enzymes (CE5 and CE15) were further employed for an analysis of their expression levels by means of hierarchical grouping, and it was possible to observe five expression groups (Fig. 2b).

Group I contained the genes expressed at lower levels, including the GH55 endo-1,3-β-glucosidase (KKO98539.1) and a subgroup consisting of a GH3 β-glucosidase H (KKP04308.1) and GH45 endoglucanase (KKO98959.1).

Group II was composed of 7 β-glucosidase, including three β-glucosidase A genes belonging to the GH3 family (KKP00605.1, KKP05725.1, and KKP05758.1), a β-glucosidase F from the GH3 family (KKP01385.1), a β-glucosidase E from the GH3 family (KKO97125.1) and a β-glucosidase B from the GH5 family (KKP05604.1).

Group III was formed by two subgroups. Subgroup III.a was composed of an endoglucanase II from the GH5 family (KKP03485.1) and a β-glucosidase M from the GH3 family (KKO97043.1), while subgroup III.b was composed of an endo-1,3-β-glucosidase M from the GH17 family (KKP05999.1) and a glucan-1,3-β-glucosidase from the GH55 family (KKP03210.1).

Group IV consisted of an esterase with a CBM1 module from the CE5 family (KKP06491.1), an α-glucosidase from the GH31 family (KKP03969.1), a 1,3-β-glucosidase from the GH17 family (KKP05186.1), a methylesterase from the CE15 family (KKO96622.1), an endoglucanase from the GH45 family (KKP04958.1), a β-glucosidase 3A from the GH3 family (KKP07011.1) and an endoglucanase from the GH5 family (KKO98175.1).

Group V consisted of two genes: an endoglucanase from the GH7 family (KKP04531.1) and an α-1-arabinofuranosidase from the GH62 family (KKP03811.1).

A β-glucosidase 1B gene from the GH1 family (KKO98105.1), a cellobiohydrolase from the GH6 family (KKP03494.1) and an AA9/GH61 protein with a CBM1 module (KKP05760.1) were not grouped.

### Phylogenetic analysis and functional diversity

**Fig. 3:**
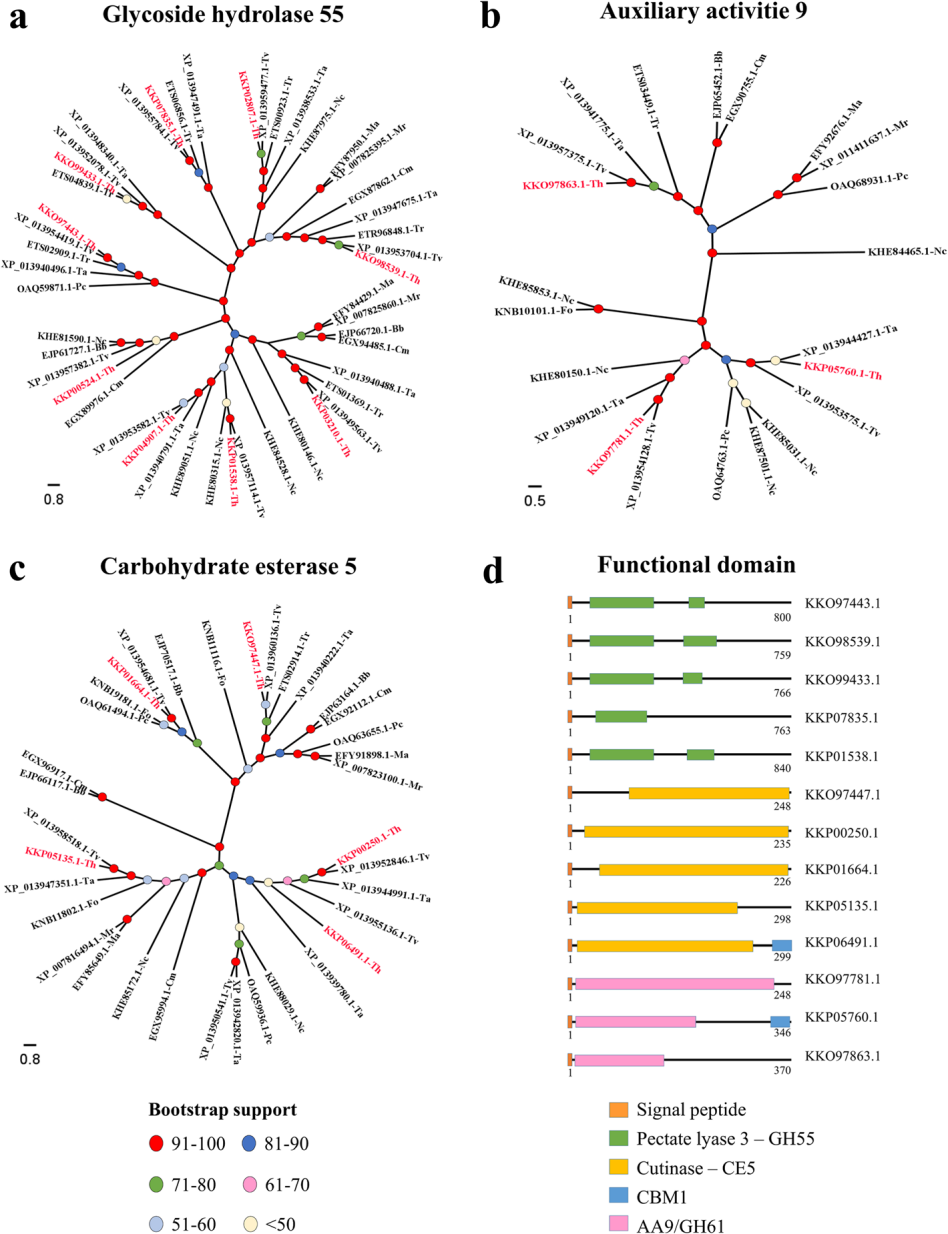
Phylogenetic tree of the GH55, AA9/GH61 and CE5 families. The tree includes the GH55 (**a**), AA9/GH61 (**b**)**,** and CE5 (**c**) proteins from twelve species and the predicted functional domains of the major proteins of the families in *T. harzianum* (**d**). *T. harzianum* proteins are highlighted in red in the tree

A phylogenetic analysis was performed to study the functional diversity of the three CAZyme families from *T. harzianum* (GH55, AA9/GH61 and CE5) and nine other species of fungi. The GH55 family was formed by nine glucan 1,3-β-glucosidase proteins in *T. harzianum* (identified as: KKO97443.1, KKO99433.1, KKP07835.1, KKO98539.1, KKP02807.1, KKP03210.1, KKP01538.1, KKP04907.1 and KKP00524.1). Most of the *T. harzianum* GH55 family proteins formed a separate clade with species from the same genus, including *T. reesei*, *T. atroviride* and *T. virens*. However, KKP00524.1 formed an external group that was closest to the GH55 of *Cordyceps militaris* (EGX89976.1) (Fig. 3a).

The AA9/GH61 family (Copper-dependent lytic polysaccharide monooxygenases – LPMOs) was formed by three proteins in *T. harzianum*, KKO97863.1, KKP05760.1 and KKO97781.1, and it formed a separate clade with other AA9 proteins from *T. reesei*, *T. atroviride* and *T. virens*. KKO97781.1 was grouped with a *T. virens* AA9 (XP_013954128.1) protein with 92% bootstrap support (Fig. 3b).

The CE5 family was formed by five proteins in *T. harzianum*, including two cutinase (KKP01664.1 and KKO97447.1) and three acetylxylan esterases (KKP00250.1, KKP06491.1 and KKP05135.1). All *T. harzianum* CE5 proteins were grouped with *T. virens* proteins throughout the tree. Wide phylogenetic diversity was observed among the CE5 family of *T. harzianum*, distributed in nearly all clades of the phylogenetic tree (Fig. 3c).

**Fig. 4:**
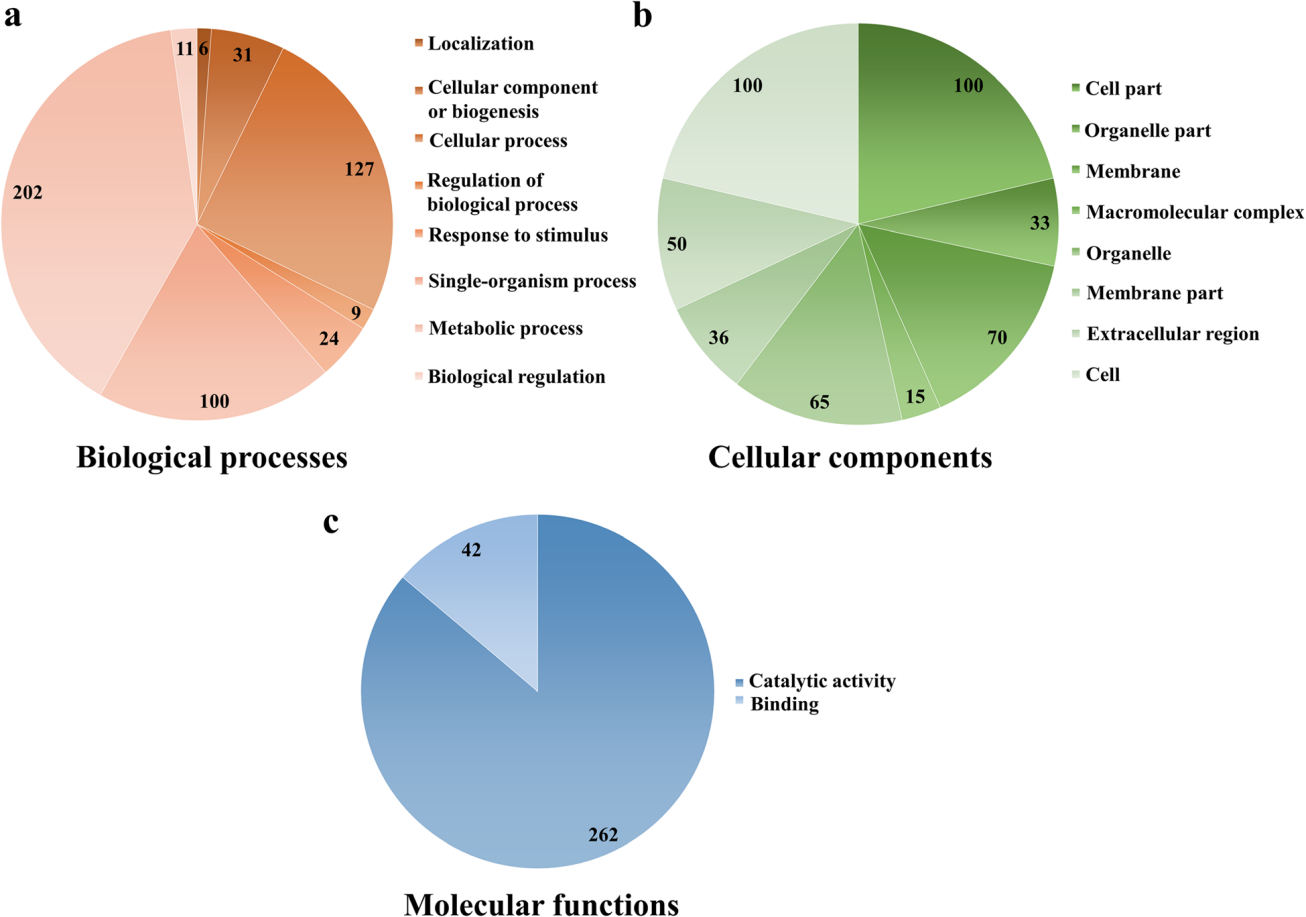
GO terms for the CAZymes of *T. harzianum*. The sequences were annotated according to three main GO terms: biological process (**a**), cellular components (**b**)**,** and molecular functions (**c**)

Functional diversity was also analyzed using the functional domains and ontologies. The functional domains validated the CAZyme classifications and confirmed the characteristic module of each family (Fig. 3d and Additional file 8: Table S3**)**. A total of 281 sequences were annotated with gene ontology (GO) terms (Fig. 4), and all of the proteins presented correspondence with the InterPro database (Additional file 9: Table S4). Catalytic activity was the main function under the molecular function term with 262 annotated sequences, while for the biological process term, the main functions were metabolic processes (202 sequences) and cellular processes (127 sequences).

## Discussion

In this study, we performed a comprehensive analysis of the total content of the CAZymes in *T. harzianum* T6776, which is widely used in biologic control and has recently been the focus of studies related to enzymes involved in the degradation of vegetal biomass. Furthermore, we performed a comparison with other *Trichoderma* spp. (*T. reesei*, *T atroviride* and *T. virens*), completed an expression analysis via RNA-Seq and explored functional diversity through a phylogenetic analysis. Thus, we present the most complete report on the total CAZymes annotated for the cellulolytic fungus *T. harzianum*.

Until recently, most studies involving *T. harzianum* were restricted to investigating its high biocontrol capacity [5, 34]. However, our research group has performed many studies evaluating the biotechnological potential of this species. Horta et al. (2014) [19] determined the transcriptome profile of this fungus during biomass degradation and delimited groups of overexpressed genes. Crucello et al. (2015) [35] constructed the first bacterial artificial chromosome (BAC) library and found a cluster of CAZymes that were probably co-expressed, and Santos et al. (2016) [36] heterologously expressed a GH1 beta-glucosidase from *T. harzianum* in *Escherichia coli* and characterized its structure and function.

Several previous studies have described the contents of CAZymes in bacteria [37, 38], fungi [39–41] and plants [42, 43]. We identified a large number of CAZymes in *T. harzianum*, *T. reesei*, *T. atroviride* and *T. virens* (430, 355, 441 and 422 CAZymes, respectively), with a high diversity of families (Fig. 1 and Table 1). These results demonstrate the importance and complexity of the system involved in the degradation of vegetal biomass that has developed in these fungi over evolutionary time [44]. The contents of CAZymes have been determined in several fungi of biotechnological importance, including *Fusarium graminearum*, *Aspergillus nidulans*, *Neurospora crassa* and *A. niger,* and 501, 441, 311 and 518 CAZy proteins were identified, respectively (CAZy database [14]).

*T. harzianum* presented 259 GHs, a number that is greater than that found in *T. reesei* RUT C-30 (198 GHs) (Table 1). Although *T. reesei* presents a lower CAZyme content than *T. harzianum,* its high efficiency in the production of hydrolytic enzymes has been demonstrated in several studies [45, 46]. This result indicates that the number of enzymes is not related to the efficiency of the biomass degradation process. In addition, previous studies in which genomic comparisons of *Trichoderma* species were performed confirmed that *T. reesei* has suffered events involving loss of CAZy genes [44, 47]. The number of CAZymes identified in *T. reesei* RUT C-30 (198 GHs, 20 CEs and 5 PLs) in our analysis was close to that found in a study in which the CAZyme content of *T. reesei* QM6a was re-annotated [39], which identified 201 GHs, 22 CEs and 5 PLs.

The CAZyme class with the greatest number of proteins among all species evaluated was the GHs. This class of enzymes is important in several metabolic routes in the fungus, including those for chitin and cellulose [48, 49]. Within this class, the most representative family was GH18, whose members are mainly involved in the degradation of chitin, which is an important route both biologically and economically, since they are applied in the biological control [50]. Another very representative family in the analysis was GH3, including enzymes showing beta-glucosidase and xylosidase activities, which are important in the metabolism of glucose and xylose [51], respectively.

We analyzed the expression of the 430 CAZymes of *T. harzianum* by mapping previously obtained RNA-Seq data from three biological conditions (with cellulose, lactose or sugarcane bagasse as a carbon source) [19]. In total, 243 GHs were expressed in the cellulose condition, which was expected because a high proportion of the enzymes that act in the degradation of biomass are from the GH family [52]. In an experiment using *T. harzianum* grown in the presence of sugarcane bagasse, a large number of GH5 and GH16 family members were observed [19]. The AA family also exhibited a large number of expressed genes, and 35 of the 42 total genes from this group were expressed in the cellulose condition. This finding reinforces that auxiliary enzymes are of great importance in the efficiency of the synergism of the main enzymes that act in the process of cellulose degradation [53].

The phylogenetic analysis of three CAZyme families (GH55, CE5 and AA9) from *T. harzianum* showed high functional diversity in these groups of proteins. This functional diversity may be a reflection of changes in the functional domains of these proteins that imply different biological actions and efficiencies of these enzymes in biological processes [54]. In addition, a large majority of the enzymes of *T. harzianum* formed groups with enzymes from *T. reesei*, *T. virens* and *T. atroviride*, demonstrating the phylogenetic proximity of these species. This result reinforces the hypothesis that the CAZymes of *Trichoderma* spp. evolved from a common ancestor [47].

## Conclusions

Searching for the main proteins used by *T. harzianum* in the degradation of biomass ensures new advances in the field of biofuel production. Herein, we annotated and characterized all of the CAZymes from *T. harzianum* at the expression level*.* The obtained data will contribute to future studies focusing on the functional and structural characterization of the identified proteins. We found a large variety of enzyme families that are related to the cellulose degradation process, and based on our results, groups of enzymes can be selected for testing enzymatic efficiency and characterizing functionality using heterologous expression. Through phylogenetic analysis of the three CAZyme families (AA9/GH61, CE5 and GH55), it was possible to observe high functional diversity within a given family, which may have implications in the process of choosing the most efficient enzymes.

## Additional files

Additional file 1: Protein sequence of the CAZymes in *T. harzianum* in FASTA format. (FASTA 264 kb)

Additional file 2: Protein sequence of the CAZymes in *T. reesei* in FASTA format. (FASTA 226 kb)

Additional file 3: Protein sequence of the CAZymes in *T. virens* in FASTA format. (FASTA 283 kb)

Additional file 4: Protein sequence of the CAZymes in *T. atroviride* in FASTA format. (FASTA 274 kb)

Additional file 5: Multiple sequence alignments used in the phylogenetic analysis of the GH55, AA9/GH61 and CE5 families. (PDF 1916 kb)

Additional file 6: Table S1. Mapping of the *Trichoderma* spp. proteins against the CAZy database. (XLSX 172 kb)

Additional file 7: Table S2. Mapping of RNA-Seq reads against the CAZymes of *T. harzianum*. (XLSX 121 kb)

Additional file 8: Table S3. PFAM domains of the CAZymes from *T. harzianum*. (XLSX 70 kb)

Additional file 9: Table S4. Gene ontology and InterPro domains of the CAZymes in *T. harzianum*. (XLSX 67 kb)

## Abbreviations

AA: Auxiliary enzymes
BLAST: Basic local alignment search tool
CAZymes: Carbohydrate-active enzymes
CDD: Conserved Domain Database
CE: Carbohydrate esterases
CEL: Cellulose
DSB: Delignified sugarcane bagasse
GH: Glycoside hydrolases
GO: Gene ontology
GT: Glycosyl transferases
LAC: Lactose
LP: Polysaccharide lyases
LPMOs: Cooper-dependent lytic polysaccharide monooxygenases
Ta: *Trichoderma atroviride*
Th: *Trichoderma harzianum*
TPM: transcripts per million
Tr: *Trichoderma reesei*
Tv: *Trichoderma virens*
