# Supplementary material for: Carbohydrate-active enzymes in Trichoderma harzianum: a bioinformatic analysis bioprospecting for key enzymes for the biofuels industry
Source: BMC Genomics. 2017 Oct 12;18:779. doi: 10.1186/s12864-017-4181-9 (PMC5639747; doi:10.1186/s12864-017-4181-9)
Supplement: Supplementary file 5 — Multiple sequence alignments used in the phylogenetic analysis of the GH55, AA9/GH61 and CE5 families. (PDF 1916 kb) [file 12864_2017_4181_MOESM5_ESM.pdf]

GH55

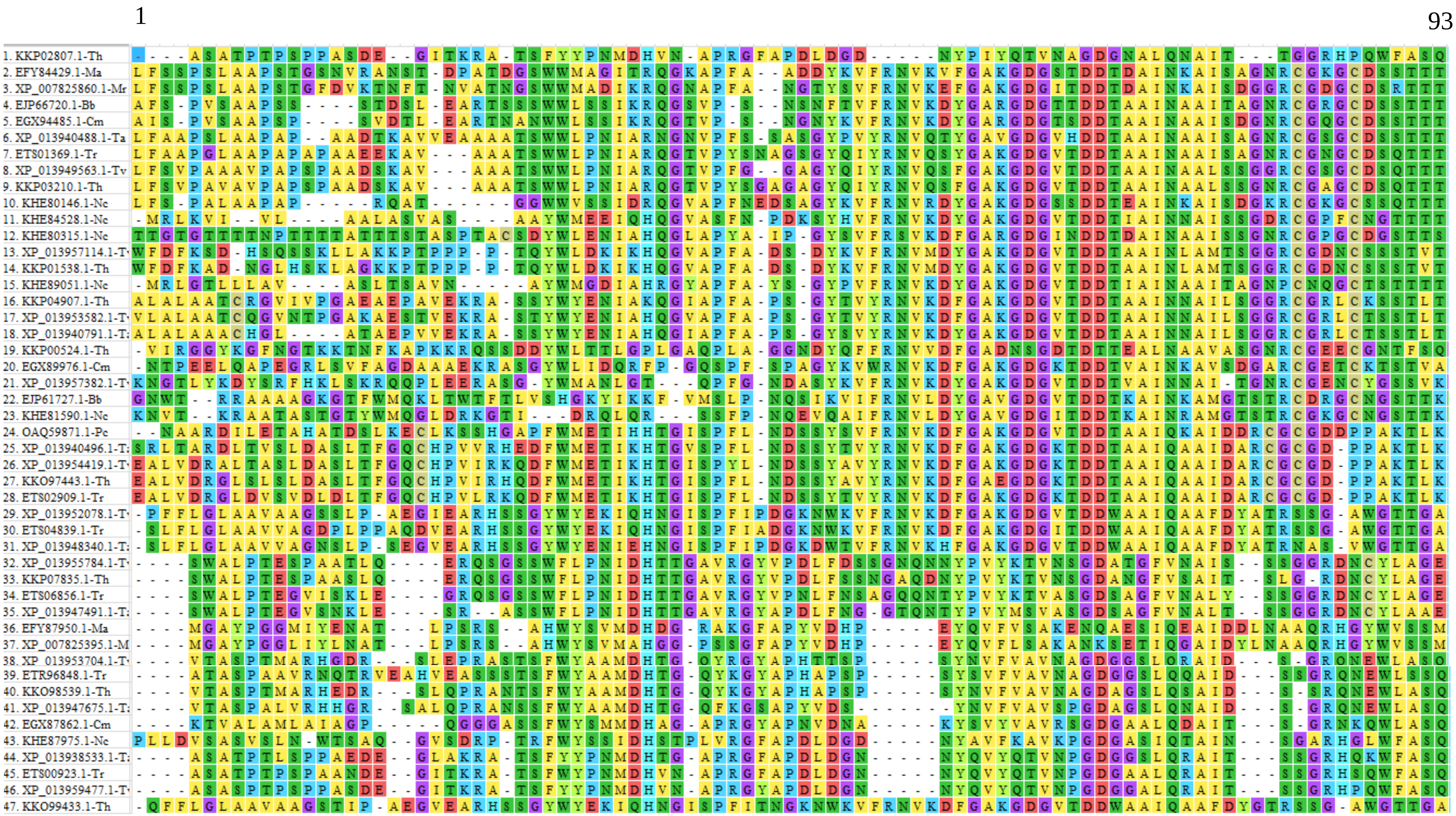
1
93
1. KKP02807.1-Th
2. EFY84429.1-Ma
3. XP_007825860.1-Mr
4. EJP66720.1-Bb
5. EGX94485.1-Cm
6. XP_013940488.1-Ta
7. ETS01369.1-Tr
8. XP_013949563.1-Tv
9. KKP03210.1-Th
10. KHE80146.1-Nc
11. KHE84528.1-Nc
12. KHE80315.1-Nc
13. XP_013957114.1-T
14. KKP01538.1-Th
15. KHE89051.1-Nc
16. KKP04907.1-Th
17. XP_013953582.1-T
18. XP_013940791.1-T
19. KKP00524.1-Th
20. EGX89976.1-Cm
21. XP_013957382.1-T
22. EJP61727.1-Bb
23. KHE81590.1-Nc
24. OAQ59871.1-Pc
25. XP_013940496.1-T
26. XP_013954419.1-T
27. KKO97443.1-Th
28. ETS02909.1-Tr
29. XP_013952078.1-T
30. ETS04839.1-Tr
31. XP_013948340.1-T
32. XP_013955784.1-T
33. KKP07835.1-Th
34. ETS06856.1-Tr
35. XP_013947491.1-T
36. EFY87950.1-Ma
37. XP_007825395.1-M
38. XP_013953704.1-T
39. ETR96848.1-Tr
40. KKO98539.1-Th
41. XP_013947675.1-T
42. EGX87862.1-Cm
43. KHE87975.1-Nc
44. XP_013938533.1-T
45. ETS00923.1-Tr
46. XP_013959477.1-T
47. KKO99433.1-Th

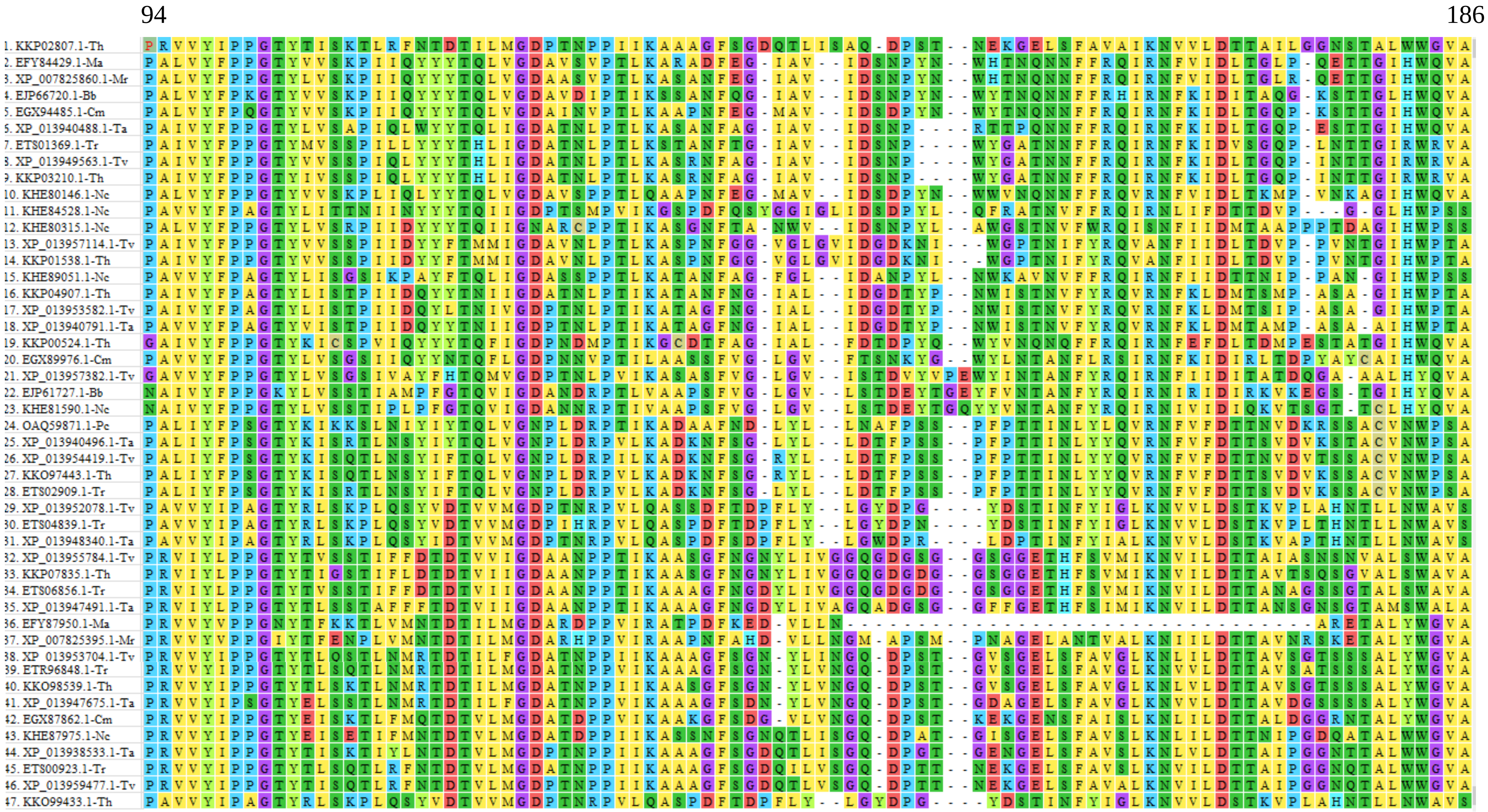
94
186
1. KKP02807.1-Th
2. EFY84429.1-Ma
3. XP_007825860.1-Mr
4. EJP66720.1-Bb
5. EGX94485.1-Cm
6. XP_013940488.1-Ta
7. ETS01369.1-Tr
8. XP_013949563.1-Tv
9. KKP03210.1-Th
10. KHE80146.1-Nc
11. KHE84528.1-Nc
12. KHE80315.1-Nc
13. XP_013957114.1-Tv
14. KKP01538.1-Th
15. KHE89051.1-Nc
16. KKP04907.1-Th
17. XP_013953582.1-Tv
18. XP_013940791.1-Ta
19. KKP00524.1-Th
20. EGX89976.1-Cm
21. XP_013957382.1-Tv
22. EJP61727.1-Bb
23. KHE81590.1-Nc
24. OAQ59871.1-Pc
25. XP_013940496.1-Ta
26. XP_013954419.1-Tv
27. KKO97443.1-Th
28. ETS02909.1-Tr
29. XP_013952078.1-Tv
30. ETS04839.1-Tr
31. XP_013948340.1-Ta
32. XP_013955784.1-Tv
33. KKP07835.1-Th
34. ETS06856.1-Tr
35. XP_013947491.1-Ta
36. EFY87950.1-Ma
37. XP_007825395.1-Mr
38. XP_013953704.1-Tv
39. ETR96848.1-Tr
40. KKO98539.1-Th
41. XP_013947675.1-Ta
42. EGX87862.1-Cm
43. KHE87975.1-Nc
44. XP_013938533.1-Ta
45. ETS00923.1-Tr
46. XP_013959477.1-Tv
47. KKO99433.1-Th

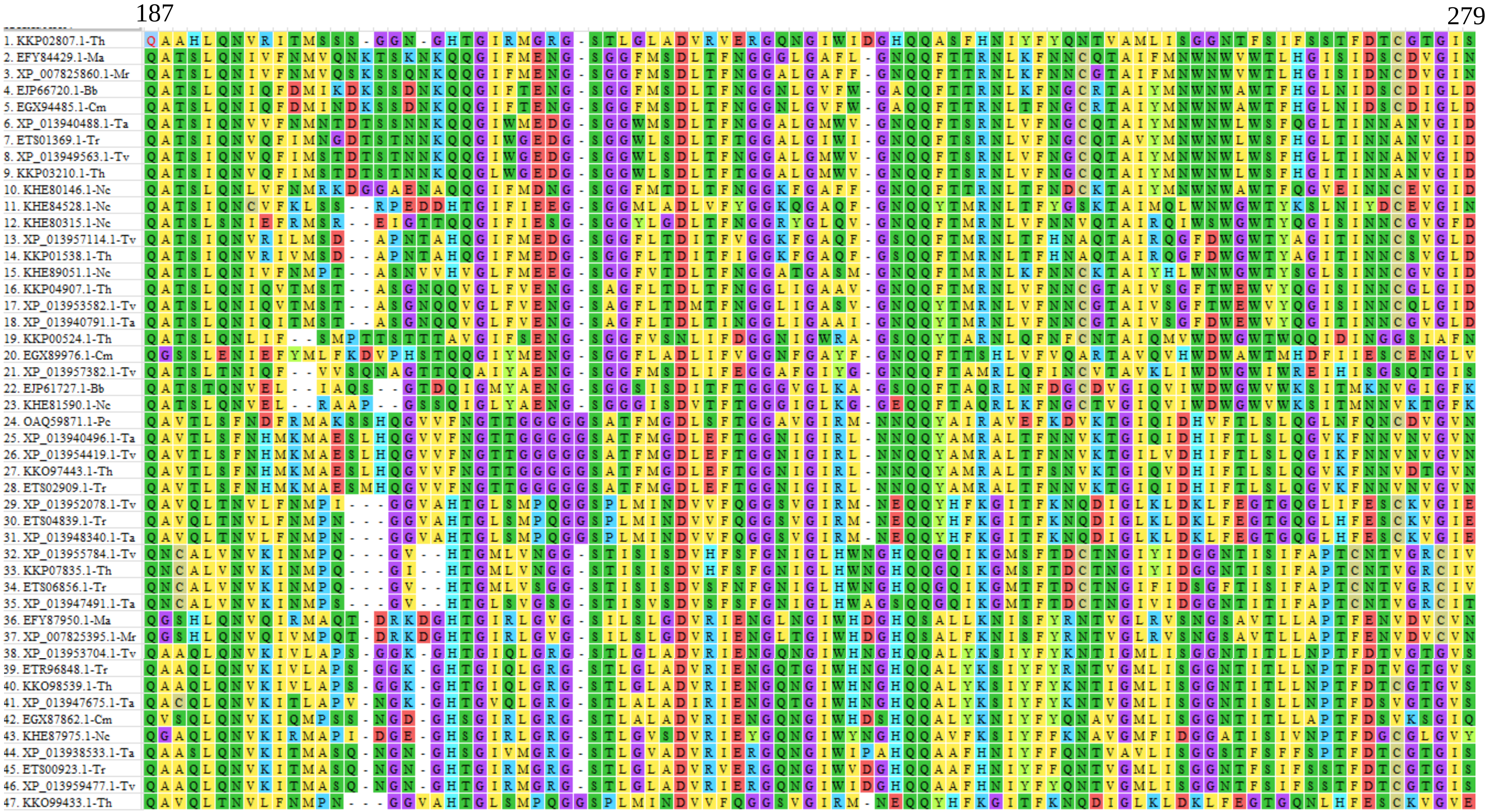
187
279
1. KKP02807.1-Th
2. EFY84429.1-Ma
3. XP_007825860.1-Mr
4. EJP66720.1-Bb
5. EGX94485.1-Cm
6. XP_013940488.1-Ta
7. ETS01369.1-Tr
8. XP_013949563.1-Tv
9. KKP03210.1-Th
10. KHE80146.1-Nc
11. KHE84528.1-Nc
12. KHE80315.1-Nc
13. XP_013957114.1-Tv
14. KKP01538.1-Th
15. KHE89051.1-Nc
16. KKP04907.1-Th
17. XP_013953582.1-Tv
18. XP_013940791.1-Ta
19. KKP00524.1-Th
20. EGX89976.1-Cm
21. XP_013957382.1-Tv
22. EJP61727.1-Bb
23. KHE81590.1-Nc
24. OAQ59871.1-Pc
25. XP_013940496.1-Ta
26. XP_013954419.1-Tv
27. KKO97443.1-Th
28. ETS02909.1-Tr
29. XP_013952078.1-Tv
30. ETS04839.1-Tr
31. XP_013948340.1-Ta
32. XP_013955784.1-Tv
33. KKP07835.1-Th
34. ETS06856.1-Tr
35. XP_013947491.1-Ta
36. EFY87950.1-Ma
37. XP_007825395.1-Mr
38. XP_013953704.1-Tv
39. ETR96848.1-Tr
40. KKO98539.1-Th
41. XP_013947675.1-Ta
42. EGX87862.1-Cm
43. KHE87975.1-Nc
44. XP_013938533.1-Ta
45. ETS00923.1-Tr
46. XP_013959477.1-Tv
47. KKO99433.1-Th

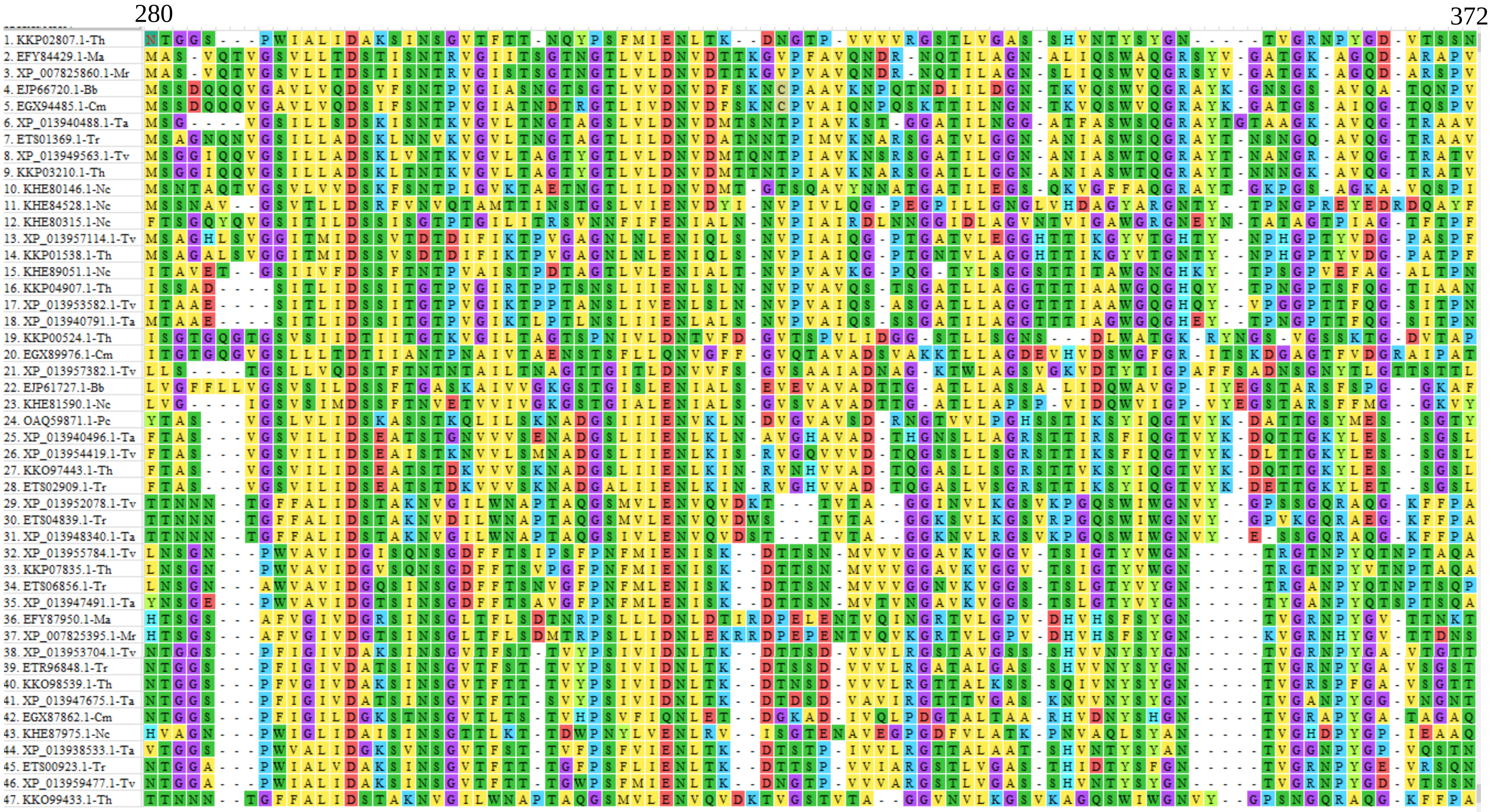
280
372
1. KKP02807.1-Th
2. EFY84429.1-Ma
3. XP_007825860.1-Mr
4. EJP66720.1-Bb
5. EGX94485.1-Cm
6. XP_013940488.1-Ta
7. ETS01369.1-Tr
8. XP_013949563.1-Tv
9. KKP03210.1-Th
10. KHE80146.1-Nc
11. KHE84528.1-Nc
12. KHE80315.1-Nc
13. XP_013957114.1-Tv
14. KKP01538.1-Th
15. KHE89051.1-Nc
16. KKP04907.1-Th
17. XP_013953582.1-Tv
18. XP_013940791.1-Ta
19. KKP00524.1-Th
20. EGX89976.1-Cm
21. XP_013957382.1-Tv
22. EJP61727.1-Bb
23. KHE81590.1-Nc
24. OAQ59871.1-Pc
25. XP_013940496.1-Ta
26. XP_013954419.1-Tv
27. KKO97443.1-Th
28. ETS02909.1-Tr
29. XP_013952078.1-Tv
30. ETS04839.1-Tr
31. XP_013948340.1-Ta
32. XP_013955784.1-Tv
33. KKP07835.1-Th
34. ETS06856.1-Tr
35. XP_013947491.1-Ta
36. EFY87950.1-Ma
37. XP_007825395.1-Mr
38. XP_013953704.1-Tv
39. ETR96848.1-Tr
40. KKO98539.1-Th
41. XP_013947675.1-Ta
42. EGX87862.1-Cm
43. KHE87975.1-Nc
44. XP_013938533.1-Ta
45. ETS00923.1-Tr
46. XP_013959477.1-Tv
47. KKO99433.1-Th

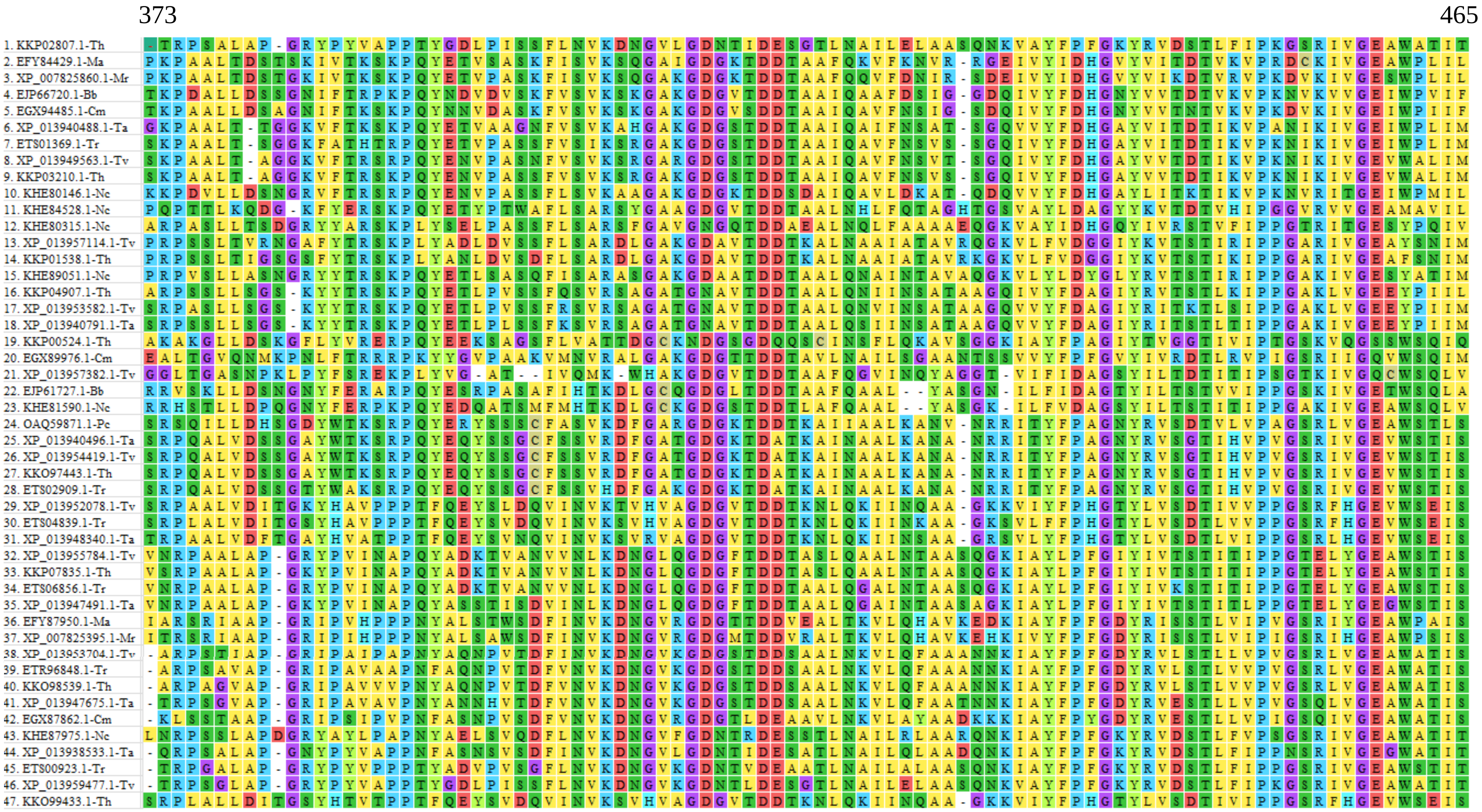

373
465
1. KKP02807.1-Th
2. EFY84429.1-Ma
3. XP_007825860.1-Mr
4. EJP66720.1-Bb
5. EGX94485.1-Cm
6. XP_013940488.1-Ta
7. ETS01369.1-Tr
8. XP_013949563.1-Tv
9. KKP03210.1-Th
10. KHE80146.1-Nc
11. KHE84528.1-Nc
12. KHE80315.1-Nc
13. XP_013957114.1-Tv
14. KKP01538.1-Th
15. KHE89051.1-Nc
16. KKP04907.1-Th
17. XP_013953582.1-Tv
18. XP_013940791.1-Ta
19. KKP00524.1-Th
20. EGX89976.1-Cm
21. XP_013957382.1-Tv
22. EJP61727.1-Bb
23. KHE81590.1-Nc
24. OAQ59871.1-Pc
25. XP_013940496.1-Ta
26. XP_013954419.1-Tv
27. KKO97443.1-Th
28. ETS02909.1-Tr
29. XP_013952078.1-Tv
30. ETS04839.1-Tr
31. XP_013948340.1-Ta
32. XP_013955784.1-Tv
33. KKP07835.1-Th
34. ETS06856.1-Tr
35. XP_013947491.1-Ta
36. EFY87950.1-Ma
37. XP_007825395.1-Mr
38. XP_013953704.1-Tv
39. ETR96848.1-Tr
40. KKO98539.1-Th
41. XP_013947675.1-Ta
42. EGX87862.1-Cm
43. KHE87975.1-Nc
44. XP_013938533.1-Ta
45. ETS00923.1-Tr
46. XP_013959477.1-Tv
47. KKO99433.1-Th

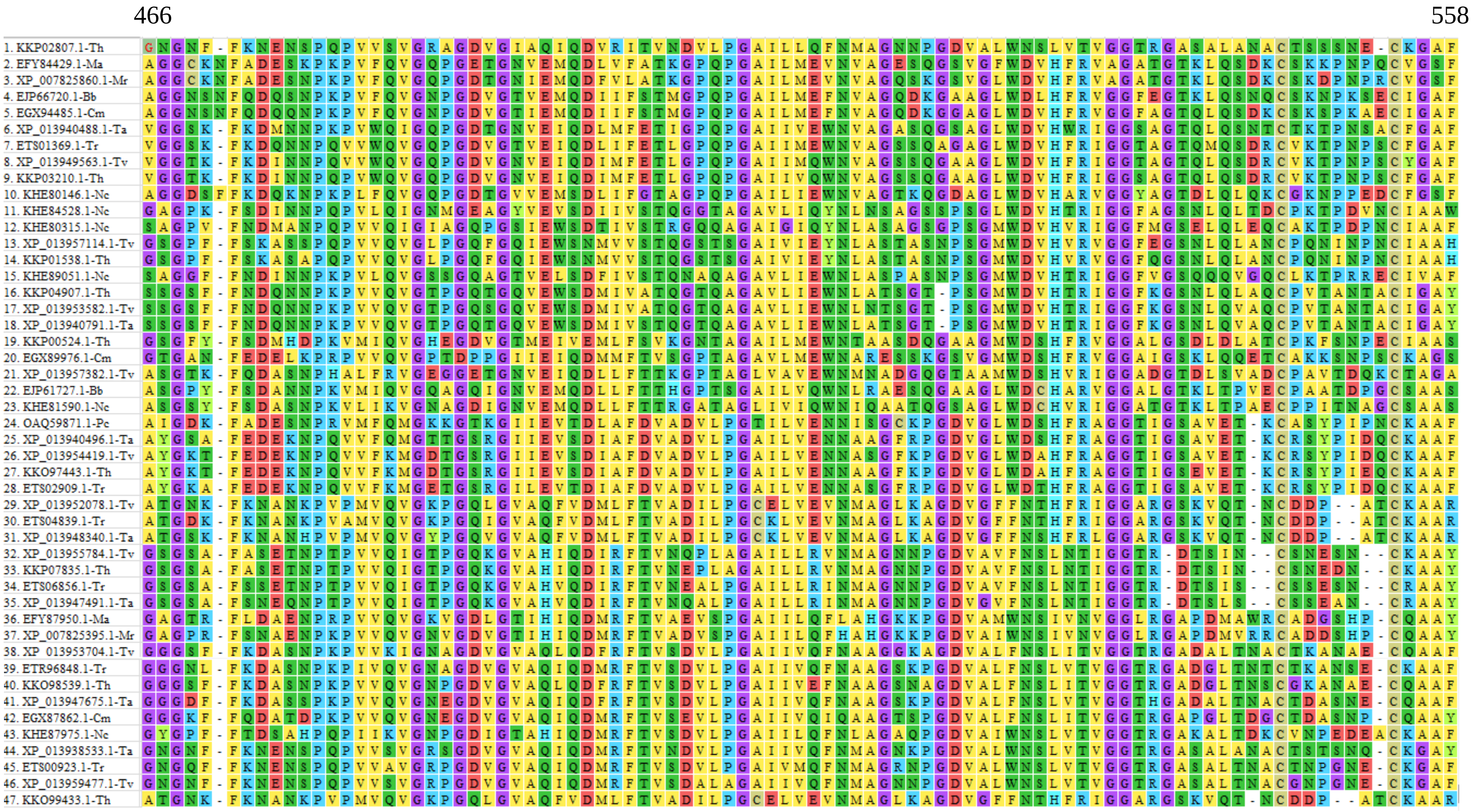
466
558
1. KKP02807.1-Th
2. EFY84429.1-Ma
3. XP_007825860.1-Mr
4. EJP66720.1-Bb
5. EGX94485.1-Cm
6. XP_013940488.1-Ta
7. ETS01369.1-Tr
8. XP_013949563.1-Tv
9. KKP03210.1-Th
10. KHE80146.1-Nc
11. KHE84528.1-Nc
12. KHE80315.1-Nc
13. XP_013957114.1-Tv
14. KKP01538.1-Th
15. KHE89051.1-Nc
16. KKP04907.1-Th
17. XP_013953582.1-Tv
18. XP_013940791.1-Ta
19. KKP00524.1-Th
20. EGX89976.1-Cm
21. XP_013957382.1-Tv
22. EJP61727.1-Bb
23. KHE81590.1-Nc
24. OAQ59871.1-Pc
25. XP_013940496.1-Ta
26. XP_013954419.1-Tv
27. KKO97443.1-Th
28. ETS02909.1-Tr
29. XP_013952078.1-Tv
30. ETS04839.1-Tr
31. XP_013948340.1-Ta
32. XP_013955784.1-Tv
33. KKP07835.1-Th
34. ETS06856.1-Tr
35. XP_013947491.1-Ta
36. EFY87950.1-Ma
37. XP_007825395.1-Mr
38. XP_013953704.1-Tv
39. ETR96848.1-Tr
40. KKO98539.1-Th
41. XP_013947675.1-Ta
42. EGX87862.1-Cm
43. KHE87975.1-Nc
44. XP_013938533.1-Ta
45. ETS00923.1-Tr
46. XP_013959477.1-Tv
47. KKO99433.1-Th

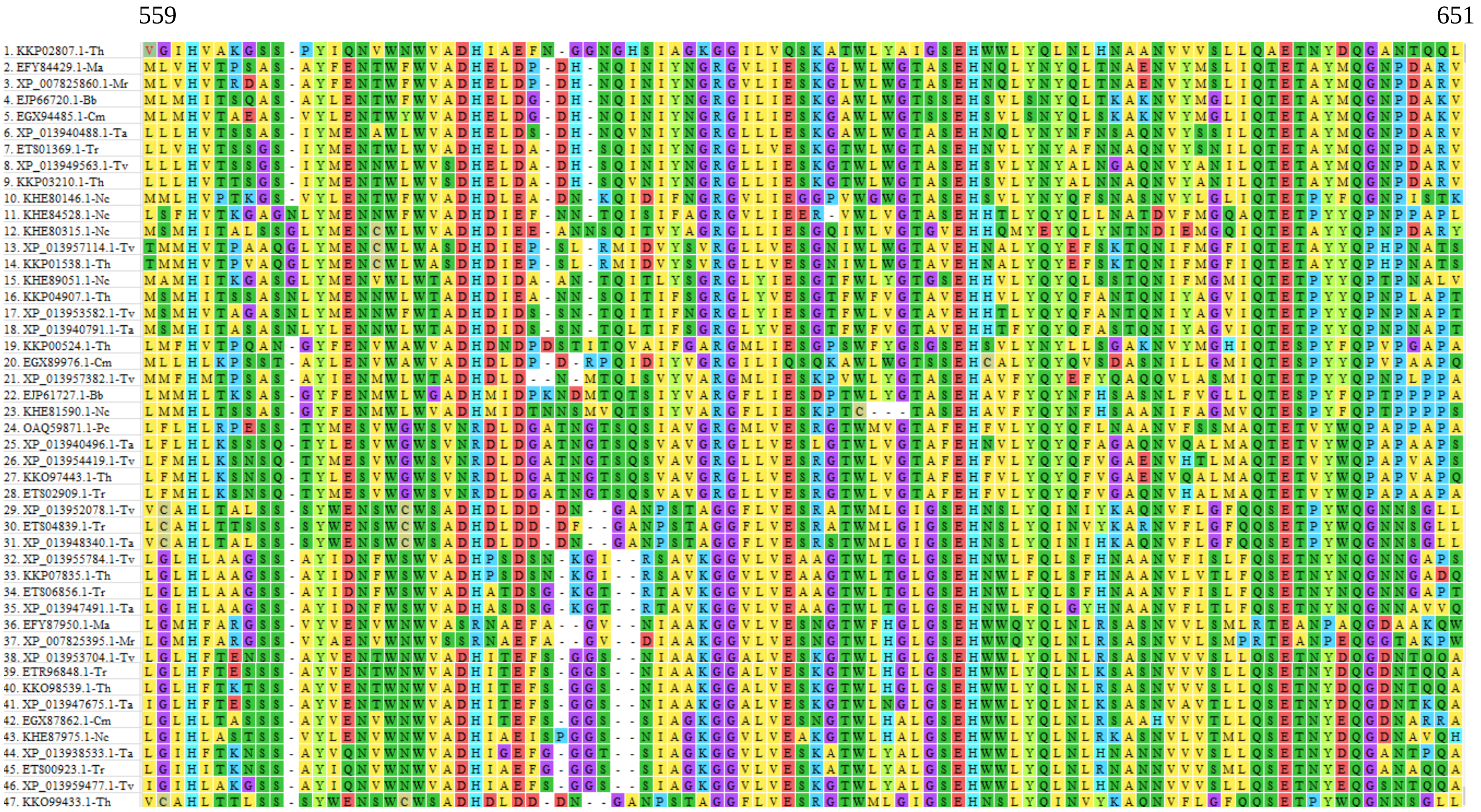
559
651
1. KKP02807.1-Th
2. EFY84429.1-Ma
3. XP_007825860.1-Mr
4. EJP66720.1-Bb
5. EGX94485.1-Cm
6. XP_013940488.1-Ta
7. ETS01369.1-Tr
8. XP_013949563.1-Tv
9. KKP03210.1-Th
10. KHE80146.1-Nc
11. KHE84528.1-Nc
12. KHE80315.1-Nc
13. XP_013957114.1-Tv
14. KKP01538.1-Th
15. KHE89051.1-Nc
16. KKP04907.1-Th
17. XP_013953582.1-Tv
18. XP_013940791.1-Ta
19. KKP00524.1-Th
20. EGX89976.1-Cm
21. XP_013957382.1-Tv
22. EJP61727.1-Bb
23. KHE81590.1-Nc
24. OAQ59871.1-Pc
25. XP_013940496.1-Ta
26. XP_013954419.1-Tv
27. KKO97443.1-Th
28. ETS02909.1-Tr
29. XP_013952078.1-Tv
30. ETS04839.1-Tr
31. XP_013948340.1-Ta
32. XP_013955784.1-Tv
33. KKP07835.1-Th
34. ETS06856.1-Tr
35. XP_013947491.1-Ta
36. EFY87950.1-Ma
37. XP_007825395.1-Mr
38. XP_013953704.1-Tv
39. ETR96848.1-Tr
40. KKO98539.1-Th
41. XP_013947675.1-Ta
42. EGX87862.1-Cm
43. KHE87975.1-Nc
44. XP_013938533.1-Ta
45. ETS00923.1-Tr
46. XP_013959477.1-Tv
47. KKO99433.1-Th

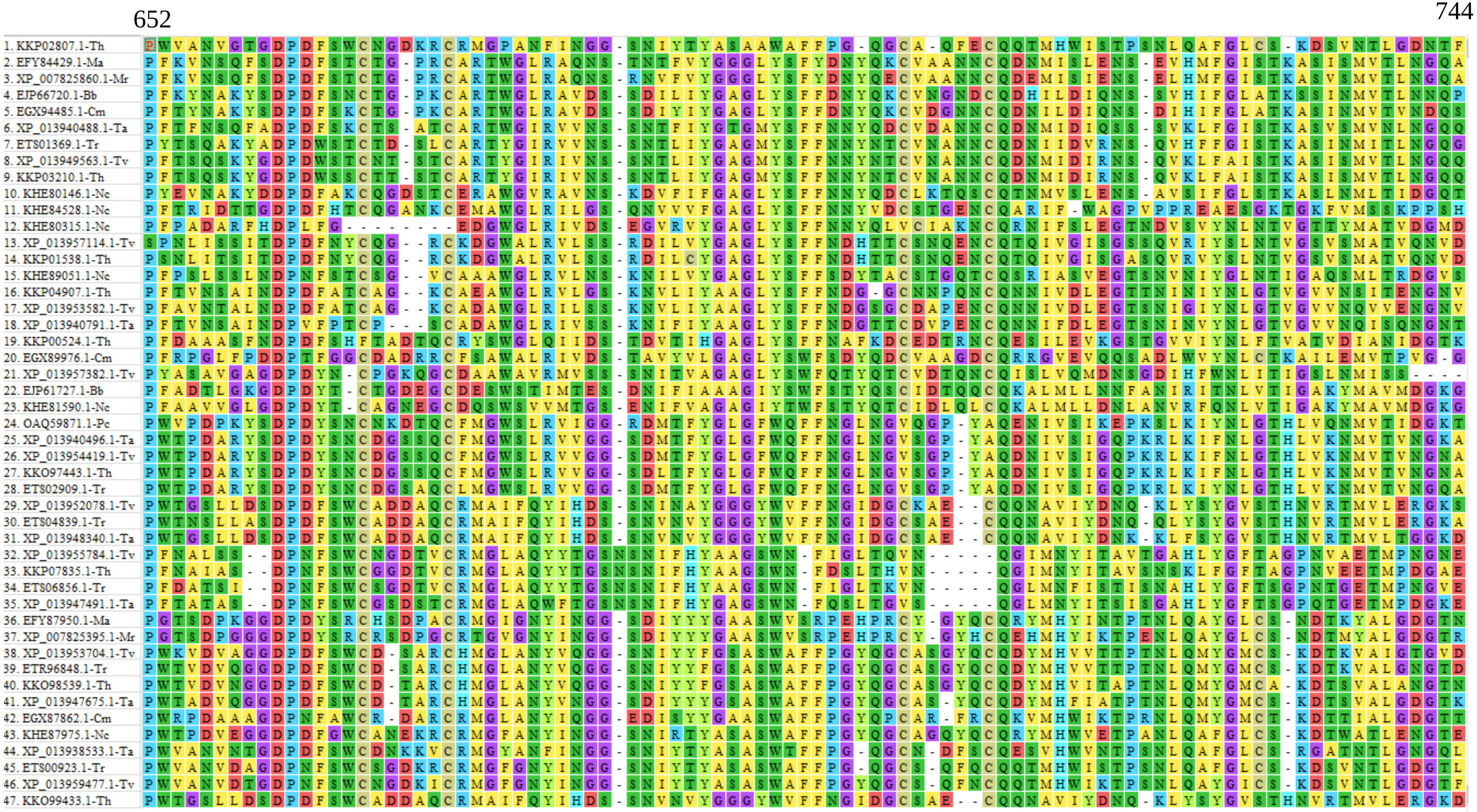
652
744
1. KKP02807.1-Th
2. EFY84429.1-Ma
3. XP_007825860.1-Mr
4. EJP66720.1-Bb
5. EGX94485.1-Cm
6. XP_013940488.1-Ta
7. ETS01369.1-Tr
8. XP_013949563.1-Tv
9. KKP03210.1-Th
10. KHE80146.1-Nc
11. KHE84528.1-Nc
12. KHE80315.1-Nc
13. XP_013957114.1-Tv
14. KKP01538.1-Th
15. KHE89051.1-Nc
16. KKP04907.1-Th
17. XP_013953582.1-Tv
18. XP_013940791.1-Ta
19. KKP00524.1-Th
20. EGX89976.1-Cm
21. XP_013957382.1-Tv
22. EJP61727.1-Bb
23. KHE81590.1-Nc
24. OAQ59871.1-Pc
25. XP_013940496.1-Ta
26. XP_013954419.1-Tv
27. KKO97443.1-Th
28. ETS02909.1-Tr
29. XP_013952078.1-Tv
30. ETS04839.1-Tr
31. XP_013948340.1-Ta
32. XP_013955784.1-Tv
33. KKP07835.1-Th
34. ETS06856.1-Tr
35. XP_013947491.1-Ta
36. EFY87950.1-Ma
37. XP_007825395.1-Mr
38. XP_013953704.1-Tv
39. ETR96848.1-Tr
40. KKO98539.1-Th
41. XP_013947675.1-Ta
42. EGX87862.1-Cm
43. KHE87975.1-Nc
44. XP_013938533.1-Ta
45. ETS00923.1-Tr
46. XP_013959477.1-Tv
47. KKO99433.1-Th

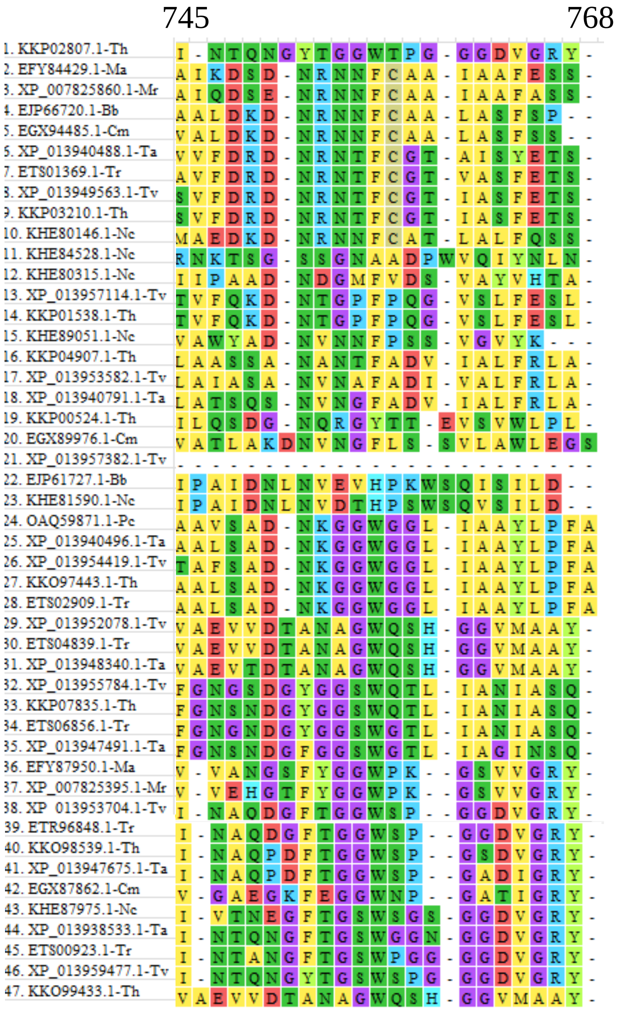
745
768
1. KKP02807.1-Th
2. EFY84429.1-Ma
3. XP_007825860.1-Mr
4. EJP66720.1-Bb
5. EGX94485.1-Cm
6. XP_013940488.1-Ta
7. ETS01369.1-Tr
8. XP_013949563.1-Tv
9. KKP03210.1-Th
10. KHE80146.1-Nc
11. KHE84528.1-Nc
12. KHE80315.1-Nc
13. XP_013957114.1-Tv
14. KKP01538.1-Th
15. KHE89051.1-Nc
16. KKP04907.1-Th
17. XP_013953582.1-Tv
18. XP_013940791.1-Ta
19. KKP00524.1-Th
20. EGX89976.1-Cm
21. XP_013957382.1-Tv
22. EJP61727.1-Bb
23. KHE81590.1-Nc
24. OAQ59871.1-Pc
25. XP_013940496.1-Ta
26. XP_013954419.1-Tv
27. KKO97443.1-Th
28. ETS02909.1-Tr
29. XP_013952078.1-Tv
30. ETS04839.1-Tr
31. XP_013948340.1-Ta
32. XP_013955784.1-Tv
33. KKP07835.1-Th
34. ETS06856.1-Tr
35. XP_013947491.1-Ta
36. EFY87950.1-Ma
37. XP_007825395.1-Mr
38. XP_013953704.1-Tv
39. ETR96848.1-Tr
40. KKO98539.1-Th
41. XP_013947675.1-Ta
42. EGX87862.1-Cm
43. KHE87975.1-Nc
44. XP_013938533.1-Ta
45. ETS00923.1-Tr
46. XP_013959477.1-Tv
47. KKO99433.1-Th

AA9

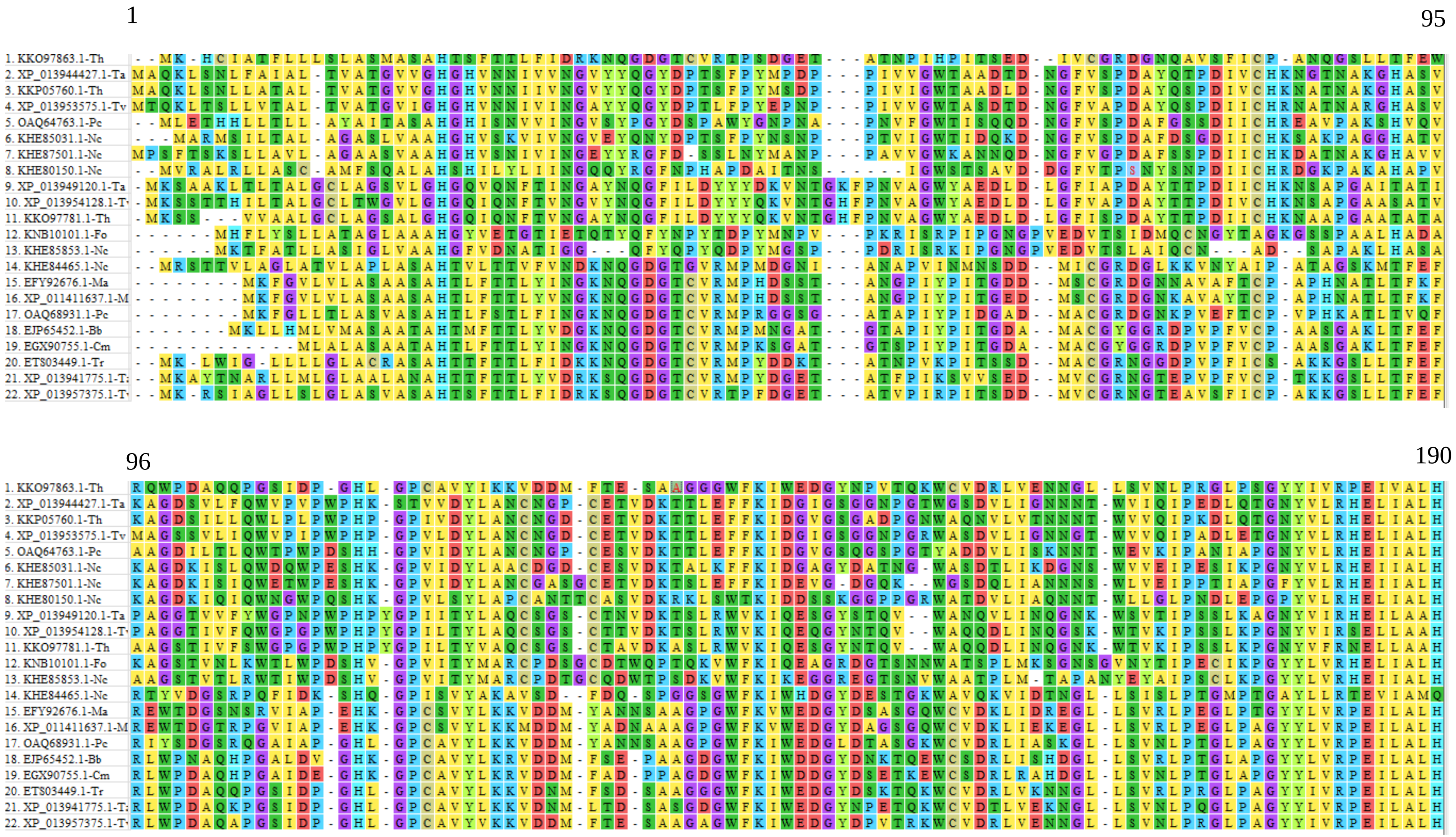

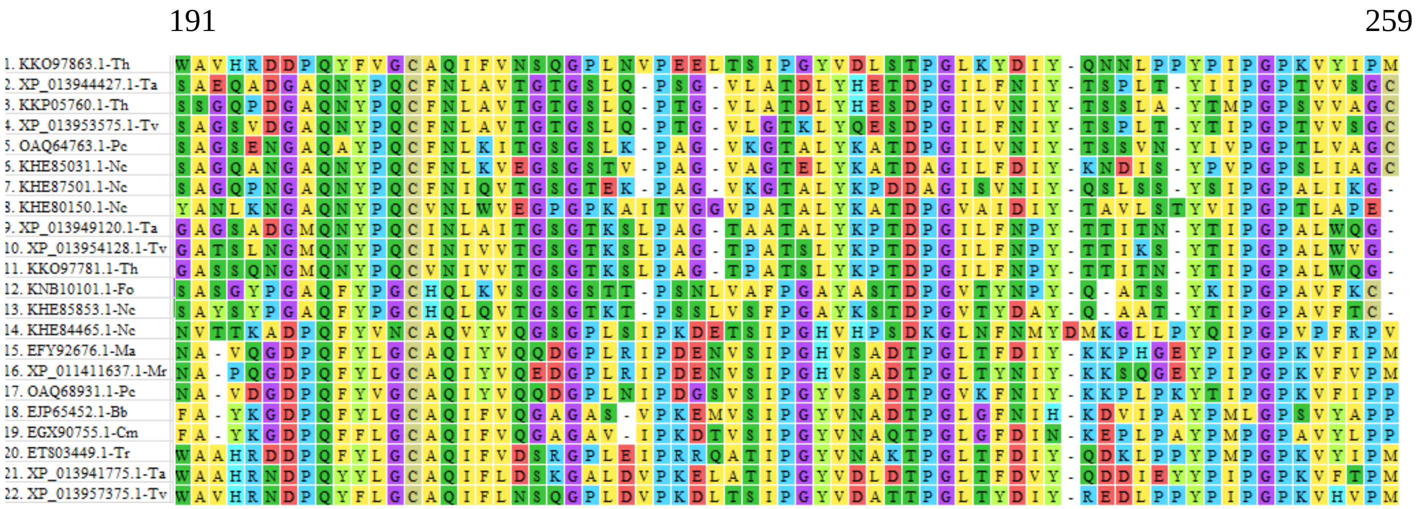
191
259
1. KKO97863.1-Th
2. XP_013944427.1-Ta
3. KKP05760.1-Th
4. XP_013953575.1-Tv
5. OAQ64763.1-Pc
6. KHE85031.1-Nc
7. KHE87501.1-Nc
8. KHE80150.1-Nc
9. XP_013949120.1-Ta
10. XP_013954128.1-Tv
11. KKO97781.1-Th
12. KNB10101.1-Fo
13. KHE85853.1-Nc
14. KHE84465.1-Nc
15. EFY92676.1-Ma
16. XP_011411637.1-Mr
17. OAQ68931.1-Pc
18. EJP65452.1-Bb
19. EGX90755.1-Cm
20. ET803449.1-Tr
21. XP_013941775.1-Ta
22. XP_013957375.1-Tv

CE5

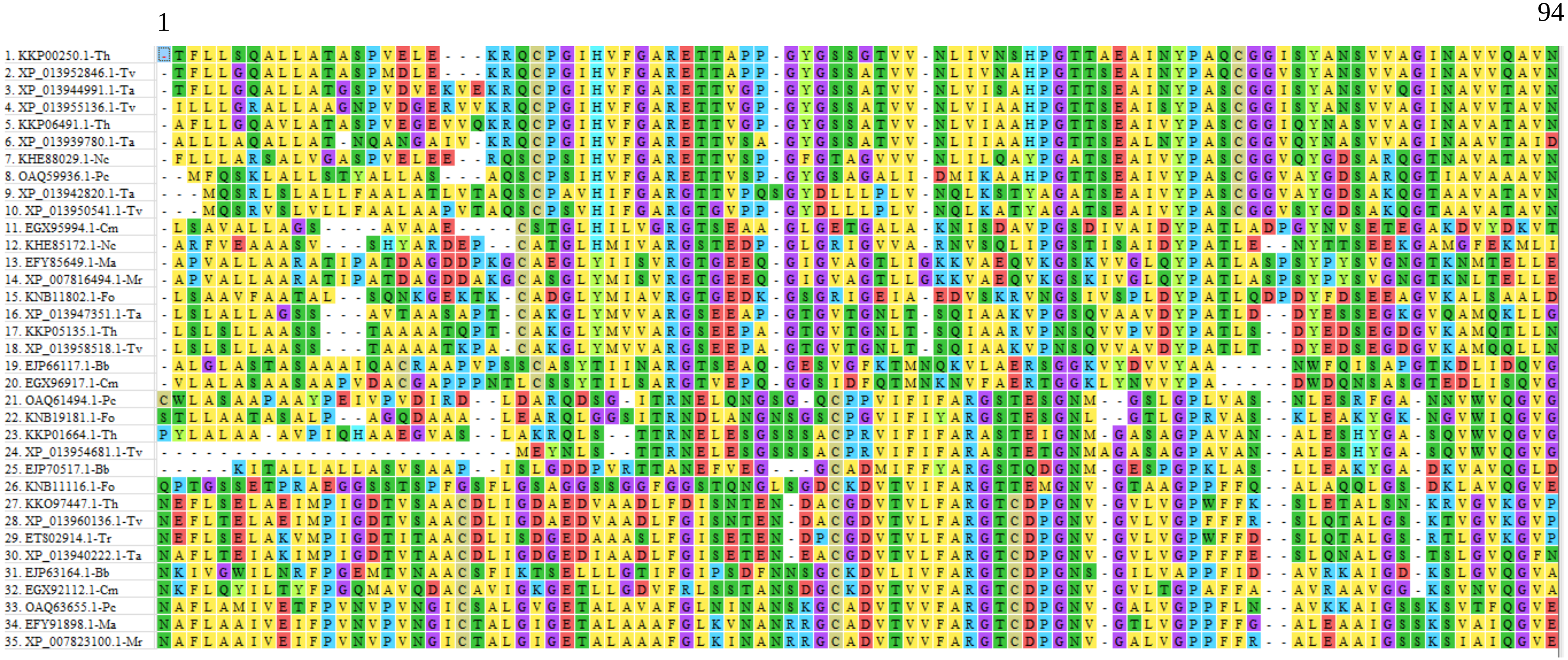
1
94
1. KKP00250.1-Th
2. XP_013952846.1-Tv
3. XP_013944991.1-Ta
4. XP_013955136.1-Tv
5. KKP06491.1-Th
6. XP_013939780.1-Ta
7. KHE88029.1-Nc
8. OAQ59936.1-Pc
9. XP_013942820.1-Ta
10. XP_013950541.1-Tv
11. EGX95994.1-Cm
12. KHE85172.1-Nc
13. EFY85649.1-Ma
14. XP_007816494.1-Mr
15. KNB11802.1-Fo
16. XP_013947351.1-Ta
17. KKP05135.1-Th
18. XP_013958518.1-Tv
19. EJP66117.1-Bb
20. EGX96917.1-Cm
21. OAQ61494.1-Pc
22. KNB19181.1-Fo
23. KKP01664.1-Th
24. XP_013954681.1-Tv
25. EJP70517.1-Bb
26. KNB11116.1-Fo
27. KKO97447.1-Th
28. XP_013960136.1-Tv
29. ETS02914.1-Tr
30. XP_013940222.1-Ta
31. EJP63164.1-Bb
32. EGX92112.1-Cm
33. OAQ63655.1-Pc
34. EFY91898.1-Ma
35. XP_007823100.1-Mr

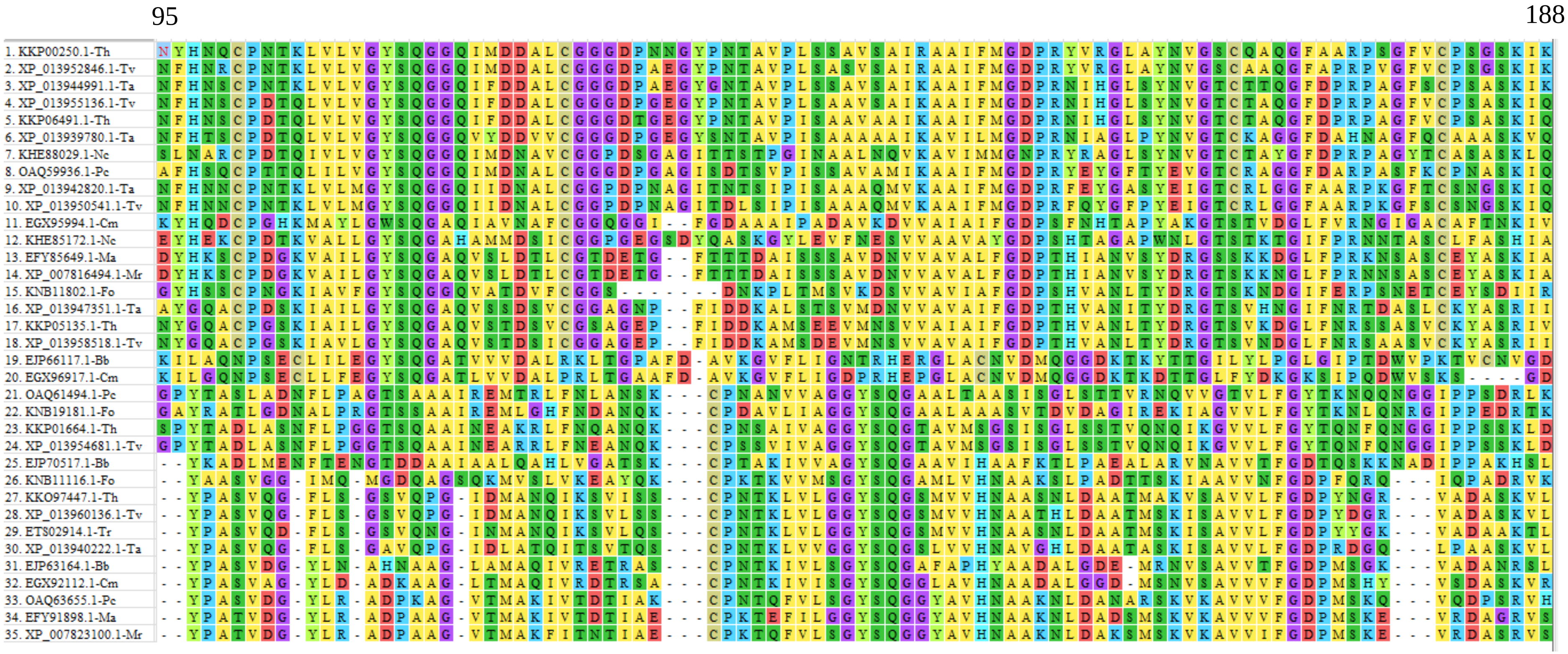
95
188
1. KKP00250.1-Th
2. XP_013952846.1-Tv
3. XP_013944991.1-Ta
4. XP_013955136.1-Tv
5. KKP06491.1-Th
6. XP_013939780.1-Ta
7. KHE88029.1-Nc
8. OAQ59936.1-Pc
9. XP_013942820.1-Ta
10. XP_013950541.1-Tv
11. EGX95994.1-Cm
12. KHE85172.1-Nc
13. EFY85649.1-Ma
14. XP_007816494.1-Mr
15. KNB11802.1-Fo
16. XP_013947351.1-Ta
17. KKP05135.1-Th
18. XP_013958518.1-Tv
19. EJP66117.1-Bb
20. EGX96917.1-Cm
21. OAQ61494.1-Pc
22. KNB19181.1-Fo
23. KKP01664.1-Th
24. XP_013954681.1-Tv
25. EJP70517.1-Bb
26. KNB11116.1-Fo
27. KKO97447.1-Th
28. XP_013960136.1-Tv
29. ETS02914.1-Tr
30. XP_013940222.1-Ta
31. EJP63164.1-Bb
32. EGX92112.1-Cm
33. OAQ63655.1-Pc
34. EFY91898.1-Ma
35. XP_007823100.1-Mr

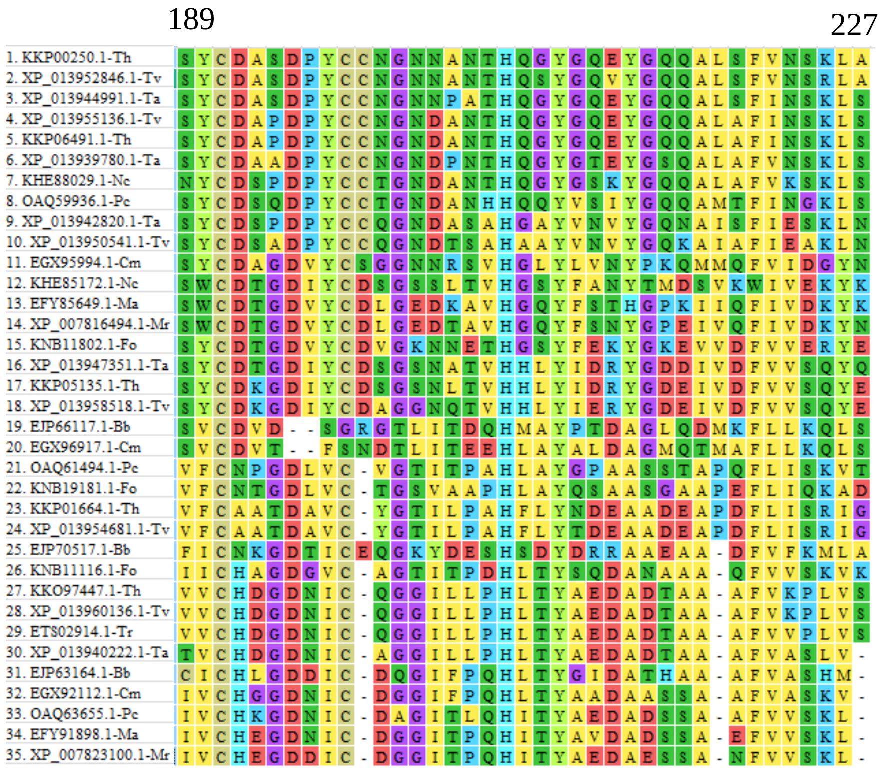
189
227
1. KKP00250.1-Th
2. XP_013952846.1-Tv
3. XP_013944991.1-Ta
4. XP_013955136.1-Tv
5. KKP06491.1-Th
6. XP_013939780.1-Ta
7. KHE88029.1-Nc
8. OAQ59936.1-Pc
9. XP_013942820.1-Ta
10. XP_013950541.1-Tv
11. EGX95994.1-Cm
12. KHE85172.1-Nc
13. EFY85649.1-Ma
14. XP_007816494.1-Mr
15. KNB11802.1-Fo
16. XP_013947351.1-Ta
17. KKP05135.1-Th
18. XP_013958518.1-Tv
19. EJP66117.1-Bb
20. EGX96917.1-Cm
21. OAQ61494.1-Pc
22. KNB19181.1-Fo
23. KKP01664.1-Th
24. XP_013954681.1-Tv
25. EJP70517.1-Bb
26. KNB11116.1-Fo
27. KKO97447.1-Th
28. XP_013960136.1-Tv
29. ETS02914.1-Tr
30. XP_013940222.1-Ta
31. EJP63164.1-Bb
32. EGX92112.1-Cm
33. OAQ63655.1-Pc
34. EFY91898.1-Ma
35. XP_007823100.1-Mr
